# Polarized or threshold training: is there a superior training intensity distribution to improve V̇O_2_max, endurance capacity and mitochondrial function? A study in Wistar Rat models

**DOI:** 10.1007/s13105-025-01079-6

**Published:** 2025-04-02

**Authors:** Pedro Oliveira, Miguel Anjos, Ariane Flores, Francisco Peixoto, Ana Isabel Padrão, Hélder Fonseca

**Affiliations:** 1 Faculty of Sport of University of Porto (FADE-UP), Research Centre in Physical Activity, Health and Leisure (CIAFEL), Porto, Portugal; 2 Laboratory for Integrative and Translational Research in Population Health (ITR), Porto, Portugal; 3 Nucleus of Research in Human Motricity Sciences, Universidad Adventista de Chile, 3780000 Chillán, Chile; 4 Vila Real Chemistry Center (CQVR), Biology and Environment Department, University of Trás-Os-Montes and Alto Douro, 5000-801 Vila Real, Portugal

**Keywords:** Polarized Training, Threshold Training, Training intensity distribution, V̇O_2_max, Mitochondrial function

## Abstract

Conflicting evidence exists regarding the superiority of Polarized Training (POL) *vs* other training intensity distribution models. Compare POL *vs* threshold (THR) training on V̇O_2_max, endurance capacity (EC) and mitochondrial function. Fifteen male Wistar rats (336.1 ± 30.4 g) were divided in: POL (*n* = 5), THR (*n* = 5) or control (CON; *n* = 5) groups. V̇O_2_max (indirect calorimetry) and EC (treadmill exhaustion test) were determined at baseline four and eight-weeks of training. POL consisted of 80% running volume at 60%V̇O_2_max and 20% at 90%V̇O_2_max while THR trained only at 75%V̇O_2_max. Both protocols were isocaloric and performed 5d/week. All animals were housed in cages with access to running wheel to allow ad libitum activity. After training, animals were sacrificed and left ventricle (LV) myocardium, diaphragm, *tibialis anterior* and *soleus* muscles were collected for high-resolution respirometry, biochemical and histological analysis. There were no baseline differences between groups. After training V̇O_2_max and EC were similar between POL and THR even though THR V̇O_2_max was higher compared to CON. After training, there were also no significant differences in OXPHOS or any of the other major mitochondrial function markers assessed between POL and THR in any of the tissues analyzed. The expression of MFN1, MFN2, PGC-1α, TFAM, DRP1, OPA1 and TOM20 as well as the activity of citrate synthase were also similar between POL and THR in all tissues. There were no significant differences in endurance performance or markers of bioenergetic function between POL and THR after eight-weeks of training.

**Supplementary Information:**

The online version contains supplementary material available at 10.1007/s13105-025-01079-6.

## Introduction

Performance in endurance sports depends highly on training intensity distribution (TID) [23, 78, 88] which can be defined according established physiological thresholds in three [79] or five zones [90]. The three-zone model defines Zone 1 (Z1) as intensity below the first ventilatory/lactate threshold, Zone 3 (Z3) above the second ventilatory/lactate threshold, and Zone 2 (Z2) between Z1-Z3 [80, 88]. Polarized (POL), Threshold (THR), Pyramidal (PYR) and High Intensity Training (HIT) are the most frequently used TIDs in endurance training [79, 88] with POL and THR being associated with the highest endurance performance improvements [40, 85, 88]. POL consists of 75–80% training in Z1, moderate percentages in Z3 (15–20%) and < 10% in Z2 [93]. THR consists of > 35% training in Z2, with the remaining distributed between Z1-Z3 [93]. In the last decade, there has been preferential adoption of POL[43] in detriment of THR [26, 44, 66, 97]. Observational studies indicate that most world-class endurance [88] and recreational runners [61] use POL as well as middle distance and long distance runners [8, 9, 48], swimmers [32] and cyclists [28], with progressive abandonment of THR TID.

Preferred adoption of THR until recently relates to the fact that Z2 training optimizes aerobic metabolism recruitment [10, 56]_._ Otherwise, POL is argued to favor mitochondrial biogenesis [19] due to stimulation of intracellular calcium signaling, potentiated by high volumes at low intensity [13, 54] and activation of 5' AMP-activated protein kinase (AMPK) signaling pathway due to cellular energy depletion by high-intensity efforts [3, 29]. POL exploits thereby the combination of low (≈80%) and high (≈20%) training intensities, optimizing the recruitment of both signaling pathways [10]. However, evidence supporting POL superiority has been questioned due to the lack of rigorously controlled studies thoroughly prescribing key training variables such as volume and intensity [7]. This limitation however could be surpassed by experimental studies in animal models [27] since laboratory conditions allow fine control and equalization of most training variables.

Our aim was to compare an eight-week POL *vs* THR training protocol on V̇O_2_max and endurance capacity (EC) in Wistar rat models. Additionally, we also investigated potential mechanisms influencing endurance performance, such as expression of mitochondrial enzymes involved in bioenergetics [17, 84], oxidative phosphorylation [49] and mitochondrial biogenesis markers in *soleus, tibialis anterior,* diaphragm and left ventricle myocardium. Our hypothesis is that POL induces superior endurance adaptations compared to THR.

## Material and methods

### Animals and experimental groups

Fifteen male Wistar rats with 4 weeks (Charles River Laboratories, France) were maintained in a 12:12 h inverted light–dark cycle, at 24 ± 2ºC, 50 ± 10% humidity with standard laboratory diet (4RF21®, Mucedola,S.r.l., Lombardia, Italy) and water ad libitum. After quarantine, rats were randomly assigned to: polarized training (POL, *n* = 5), threshold training (THR, *n* = 5) or control (CON, *n* = 5) and transferred to individual cages equipped with running wheel (Tecniplast, Buguggiate, Italy). All animals underwent afterwards a treadmill exercise training protocol for eight weeks. Food intake, running wheel activity, and body weight were monitored throughout the experiment. All procedures were performed in accordance with the guidelines for the Care and Use of Laboratory Animals in Research recommended by the European Federation of Associations for the Science of Laboratory Animals (FELASA). Local Ethics Committee approved the study protocol (CEFADE 19–2023), according to the EU directive (2010/63/EU) and Portuguese law (DL 113/2013).

### Experimental design

**Fig. 1:**
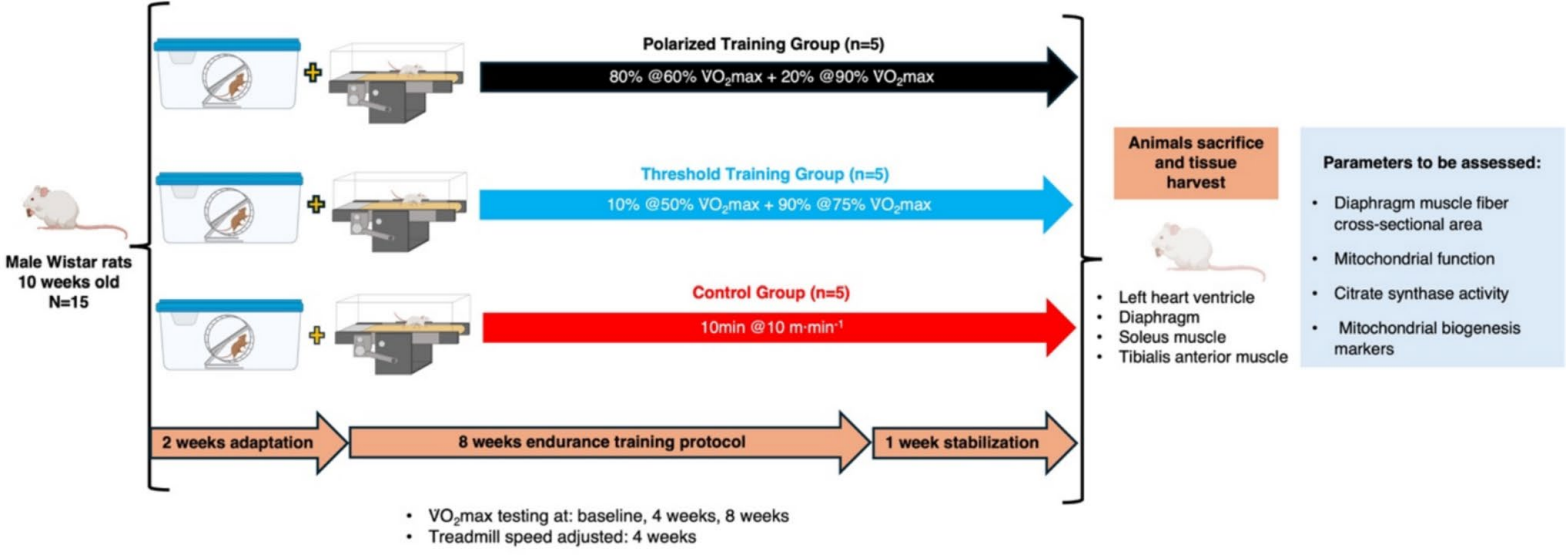
Overview of the experimental design

All animals underwent a two-week progressive treadmill (LE8710RTS multilane treadmill; Panlab Harvard Apparatus, ISA) habituation protocol after which they underwent a V̇O_2_max and EC test. V̇O_2_max was used to define training intensity during the first 4-weeks of training and was repeated halfway the protocol to readjust intensity. After the final week of training all animals underwent a stabilization week to allow recovery from potential muscle damage resulting from V̇O_2_max and EC testing (Fig. 1). All tests and exercise sessions occurred between 8–14 h to avoid bias related with fatigue and muscle glycogen concentration circadian variation.

### Exercise training protocol

The treadmill adaptation protocol lasted two weeks (10 days). On the first day (D1), rats were only placed on the immobile treadmill for 10 min to adapt to the new environment. On D2, the treadmill was turned on at 10 m·min^−1^ for 10 min. On subsequent days, speed remained constant, while duration increased 10 min/day until reaching 60 min. Afterwards, duration was kept at 60 min/day while speed increased by 2 m·min^−1^ until D10. CON group treadmill adaptation protocol included only 10 min/day at 10 m·min^−1^.

Training consisted on 5d/week sessions at 19ºC room temperature and dim lighting. All training sessions were performed at a 10º incline until week 4, after which incline increased to 15º from weeks 4 to 8. After each training animals were rewarded with 0.5 g chocolate. During exercise, animals that received > 5 shocks/min were removed from the treadmill for two min to rest being this time compensated at the end of the session.

**Fig. 2:**
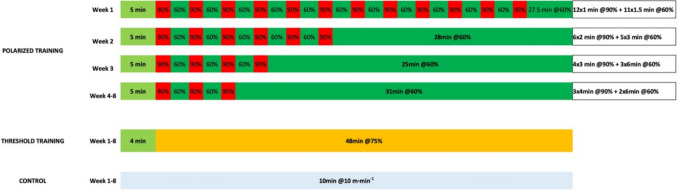
Exercise training protocol description. Different colors highlight the different exercise intensities in each group and phase of the training protocol

POL training consisted of 80% low intensity training (48 min at 60% V̇O_2_max) and 20% high intensity (12 min at 90% V̇O_2_max). For practicality, both high and low intensities occurred in the same training session. In order to enhance tolerance to high intensity training, animals performed 12 × 1 min intervals at 90% V̇O_2_max in the first week, followed by 6 × 2 min intervals at 90% (week 2), and 4 × 3 min intervals at 90% (week 3). From weeks 4 to 8, each training started with 5 min warm-up (60% V̇O_2_max), followed by 3 × 4 min intervals at 90% V̇O_2_max, interspersed with 6 min at 60% V̇O_2_max for recovery. The remaining 29 min were performed at 60% V̇O_2_max. Loads were progressed as described in Fig. 2. THR training comprised 4 min warm-up (50% V̇O_2_max) and 48 min at 75% V̇O_2_max. POL and THR protocols were isocaloric to equalize training loads. To achieve this, the duration of each training session was adjusted to ensure equal O_2_ consumption, regardless of running speed. Intensities were defined as low (60% V̇O_2_max), moderate (75% V̇O_2_max) or high (90% V̇O_2_max) according to Poole et al., classification [73]. CON group performed 10 min/day running at 10 m·min^−1^ to equalize exposure to treadmill conditions. After the 8-weeks of training, all animals underwent an additional stabilization week of training at the same intensity of the previous week before sacrifice to ensure stable training conditions and to allow recovery from any potential muscle damage that could have been elicited by V̇O_2_max and EC tests.

#### V̇O_2_max assessment protocol

V̇O_2_max was determined through a maximal treadmill exercise test (Metabolic Modular Treadmill; Columbus Instruments, Columbus, USA) with respiratory gas analysis following adequate calibration according to manufacturer instructions using primary standard grade calibration gas (Acail Gas, Portugal) and drierite columns (Calcium Sulfate with Indicator, Sigma-Aldrich; St. Louis, USA) to ensure moisture absence in the gas sample.

Before testing, animals were weighed and allowed to acclimatize for ≈10 min inside the treadmill chamber for RER stabilization. Protocol started at 10 m·min^−1^ and progressed with 3 m·min^−1^ increments every 2 min at 10° inclination. Protocol was terminated when animals remained > 5 s in the treadmill rear. V̇O_2_max was determined as the highest O_2_ consumption reached during the test. Measurements were considered valid if RER was > 1.0 and [blood lactate] > 6 mM determined in a tail vein capillary blood sample (Lactate Pro 2). Intermediate V̇O_2_max assessment at 4-weeks was modified to prevent test duration from being excessively long and final treadmill speed excessively high by setting inclination at 15^o^ and initial speed at 16 m/m followed by 6 m·min^−1^ increments in steps 2 and 3 and of 3 m·min^−1^ afterwards. The same protocol was used in the final V̇O_2_max assessment at 8 weeks.

#### EC capacity assessment protocol

EC was determined by a treadmill test starting with 5 min warm-up at 10 m·min^−1^, followed by 5 m·min^−1^ speed increments every five minutes until exhaustion (10° incline). Criteria for determining exhaustion were: (i) > 5 s on the electrified grid (ii) changes in gait pattern or reduction of righting reflex. Total distance and the time to exhaustion were recorded.

### Sacrifice and necropsy

Sacrifices occurred 48 h after last training session by an overdose with Ketamine (Nimatek 100 mg.mL^−1^, Dechra) and Xylazine (Rompun 2%). Samples were immediately collected, cleared of blood and surrounding tissue, weighted and either stored at −80 °C or immediately prepared for analysis according to each specific protocol (high-resolution respirometry, histology or biochemistry). The following samples were collected: left ventricle myocardium, diaphragm, *soleus*, and *tibialis anterior* muscles.

### Histological analysis of the diaphragm

Diaphragm muscle fiber cross sectional area was determined by histological analysis in a ≈5 mm^2^ sample taken from the anterior *diaphragm* region. Fixation was immediately carried by diffusion in 4% w/v buffered formaldehyde (252931.1315, PanReact AppliChem,) during 48 h, followed by dehydration in graded ethanol (E/0650DF/C17, Fisher Chemical) solutions, clearing in xylene (6615, Thermo Scientific) and inclusion in paraffin (8336, Thermo Scientific). Transverse 5 µm sections were obtained with a rotary microtome (Leica RM 2125, RT, Leica Microsystems; Nussloch, Germany) and mounted on silane-coated slides. After drying, slides were dewaxed in xylene, rehydrated in graded ethanol concentrations, stained with hematoxylin (Sigma-Aldrich: HHS32) and eosin (Sigma-Aldrich: HT110132), mounted with DPX (Sigma-Aldrich: 06522) and analyzed under a light microscope (Carl Zeiss Imager A1) with digital camera (AxioCam 503 color; Göttingen, Germany). Representative photomicrographs were taken and analyzed with ImageJ (NIH, Bethesda, MD, USA). Fiber cross-sectional area was determined as the average area of ≈250 fibers of each animal.

### High-resolution respirometry—OROBOROS

Approximately 5 mg of left ventricle and skeletal muscles (*soleus*, *tibialis anterior* and diaphragm) were collected immediately after sacrifice and homogenized with a PBI-Shredder O2K (Oroboros Instruments, Innsbruck, Austria) previously cooled at −20 °C as described elsewhere [20]. Samples were than prepared by adding ice-cold respiration medium, distributed on the Lysis Disk and homogenized using the SG3 Driver in a two-step shredding process. Homogenate was then transferred to a 50 mL tube, rinsed with fresh respiration medium and added to the respirometry chamber containing 2 mL of mitochondrial respiration medium MiR05 (ID: 60,101–01 Lot.20J01923, Oroboros Instruments, Innsbruck, Austria) preequilibrated at 37 °C for determination of mitochondrial respiration (Oroboros Instruments, Innsbruck, Austria). After ≈40 min calibration, the Substrate Uncoupler Inhibitor Titration protocol 1 (SUIT-001 O2 mt D001) protocol (Appendix S1 of the ESM) for O2k-sV-Module was performed.

SUIT-001 protocol reflects maintenance of flux with the addition of complex I substrates pyruvate (Sigma-Aldrich: P2256) and malate (Sigma-Aldrich: M1000) (PM), followed by a saturating amount of ADP [Merck (Calbiochem): 117,105-1GM]. Cytochrome C (Sigma-Aldrich: C7752) was added to test mitochondrial inner membrane integrity. Progressive titration of two steps of Carbonyl Cyanide-m-Chlorophenylhydrazone (CCCP; Sigma-Aldrich: C2759) was subsequently performed until no further stimulation was observed (i.e. maximal stimulation was reached). Glutamate (Sigma-Aldrich: G1626) oxidized in the mitochondrial matrix by glutamate dehydrogenase to α-ketoglutarate, representing the control state of the glutamate-anaplerotic pathway. Complex II substrate succinate (Sigma-Aldrich: S2378) was then added to stimulate mitochondrial respiration powered by both complexes I and II. Rotenone (Sigma-Aldrich: R8875), is a complex I inhibitor and thus inhibits NADH oxidation. The glycerophosphate pathway is a level 3 ETC pathway control state, supported by glycerophosphate (Gp; Santa Cruz Biotechnology: sc-215789) fuel substrate and electron transfer through the glycerophosphate dehydrogenase complex to Q function. Then, mitochondrial respiration was completely inhibited using the complex III inhibitor antimycin A (Sigma-Aldrich: A8674). Lastly, the maximal activity of complex IV was assessed using ascorbate (Sigma-Aldrich: A7631) and TMPD (Sigma-Aldrich: T3134). While there is a known consumption of chemical O_2_ linked to the auto-oxidation of ascorbate and TMPD, the consumption of chemical O_2_ was subtracted from the ascorbate and TMPD oxygen specific flux, as demonstrated in the results for the normalization of complex IV.

### Tissue homogenization and total protein quantification

Approximately 50 mg of muscle was used for the homogenization step. Homogenization was carried in ice-cold RIPA buffer (20–188, EMD Milipore) with protease (Sigma: P8340) and phosphatases inhibitors (Sigma: P0044 and P5726) using a Teflon pestle and glass Potter–Elvehjem. Total protein content was determined with *DC Protein Assay* (5,000,111, Bio-Rad®) following manufacturer protocol, using BSA as standard. Absorbance was measured at 750 nm in a microplate reader (Multiskan GO, Thermo Fischer Scientific®, Northumberland, UK).

### Western blot analysis

20 µg of each sample were separated by electrophoresis on 10 and 12.5% SDS-PAGE gels prepared according to Laemmli [50], transferred into a nitrocellulose membrane (Whatman®, Protan®, Merk) with TURBO transblot (Bio-Rad, Hercules, CA, USA) at 2500 mA, 25 V for 7 min (Mixed MW) blocked 10 min with EveryBlot Blocking Buffer (#12010020, Bio-Rad) and incubated with adequate primary antibody [rabbit polyclonal anti-mtTFA (sc-28200) 1:200; mouse monoclonal anti-Mfn2/Mitofusin 2 (F-5) (sc-515647) 1:750; mouse monoclonal anti-Mfn1/Mitofusin 1 (D-10) (sc-166644) 1:750 and rabbit polyclonal anti- PGC-1α (ab54481) 1:1000]; rabbit monoclonal anti-DRP1 (Cell Signaling Technology #8570) 1:1000; mouse monoclonal anti-OPA1 (ab119685) 1:1000 and rabbit polyclonal anti-TOM20 (sc-11415) 1:200 diluted in EveryBlot Blocking Buffer overnight at 4 °C under agitation. Membranes were washed and incubated with anti-mouse (ab205719) or anti-rabbit (ab6721) IgG peroxidase secondary antibody diluted 1:5000 in EveryBlot Blocking Buffer. Bands were detected with Clarity Western ECL Substrate (Bio-Rad), digitized with ChemiDoc Imaging System (version 2.3.0.07, Bio-Rad, Hercules, CA, USA) and analyzed with Image Lab v6.1 (Bio-Rad, Hercules, CA, USA). Membranes were stained with Ponceau S as loading control [75] and digitized for transfer control (Appendix S3 of the ESM). Immunoblot bands intensity was expressed relativized to Ponceaus S staining as recommended [77].

### Citrate synthase (CS) activity

CS activity was measured in samples homogenates following Coore et al., [16]. CoASH released from the reaction of acetyl-CoA (Sigma: A2056) with oxaloacetate (Sigma: O7753) was measured by its reaction with 5,5’-dithiobis-(2-nitrobenzoic acid) (DTNB, Sigma, D8130) at 412 nm (ε = 13.6 mM-1 cm-1). Total CS activity was expressed in nmolmin^−1^ mg^−1^ protein on each sample.

### Statistical analysis

Descriptive measures [mean and standard deviation (SD)] were determined for all relevant variables. Distribution was assessed for normality and homogeneity of variance with Shapiro–Wilk and Levene test, respectively. Whenever assumptions for parametric testing were met, comparisons for baseline values were performed by one-way ANOVA. Multiple post-hoc comparisons were performed with Bonferroni or Games-Howell test, if homogeneity of variance was present or not, respectively. Whenever variables lacked normal distribution, comparisons were performed with bootstrapping (1000 bootstrap samples). A two-way ANOVA was performed to determine the effect of the independent variable (training regimen), the effect of time and their interaction (training regimen*time effect). No outliers were removed unless stated otherwise. For this purpose, outliers were identified as data points that were i) deviated ≥ 3 standard deviations (SD) from the mean, ii) clearly isolated from the rest of the dataset, and iii) that after removal did not lead to the appearance of other outliers. Statistical analysis was performed with IBM SPSS Statistics V27.0 (Armonk, NY: IBM Corp) and graphs were assembled with GraphPad Prism (version 10.4.1, Boston, MA). Sample size was determined a priori with G*Power being established that a sample size of 15 animals (5 per group) would allow detecting significant differences between groups in a two-way pre-post analysis with a 5% type I (α) and 20% type II error probability (β), if the effect size was large (0.5).

## Results

### Bodyweight, food intake and voluntary physical activity

**Fig. 3:**
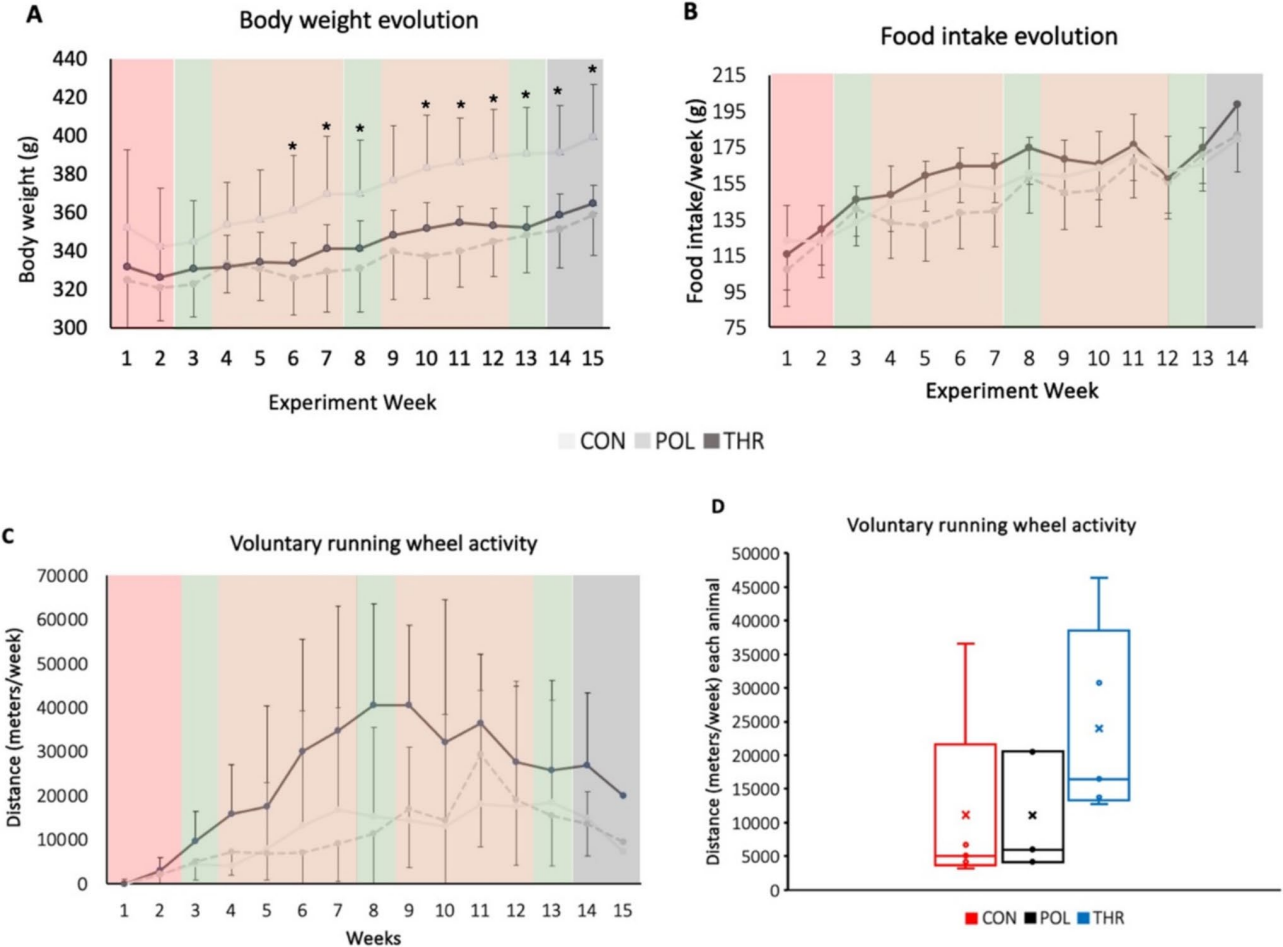
Body weight, food intake and voluntary running wheel activity. **A** Animals body weight evolution throughout the experiment; **B** average food intake per week in each group; **C** average distance traveled per week in the running wheel throughout the experiment in each group and, **D** total voluntary running wheel activity per week in each group with individual data display. Data are presented as mean ± standard deviation. CON: Control group: POL: Polarized training group; THR: Threshold training group. Experiment week 1–2 (Treadmill adaptation period – Light Red); Week 3 (V̇O_2_max and EC—Baseline tests – Light green); Week 4–7 (Exercise training protocol – Light orange); Week 8 (V̇O_2_max and EC—Mid Tests – Light green); Week 9–12 (Exercise training protocol – Light orange); Week 13 (V̇O_2_max and EC—Final Testing – Light green); Week 14 (Stabilization week—Grey); Week 15 (Euthanasia and necropsy—Grey). Asterisks highlight the existence of statistically significant differences for CON group in comparison to POL or THR (*p < 0.05)

Initially there were no weight differences between groups (*p* = 0.362). During the experiment, all groups increased body weight, but increases were lower in POL compared to CON at the end of the protocol (*p* = 0.028) (Fig. 3). However, between weeks 6 and 10, except for week 9, CON group exhibited a higher bodyweight compared to POL. From weeks 11–13, CON group consistently showed higher bodyweight compared to both POL and THR (Appendix S2 of the ESM). There were no differences in final body weight between POL and THR groups (*p* = 1.00). Average weekly food intake and running distance in the activity wheel was also similar between groups (*p* = 0.245; *p* = 0.181). Despite the lack of differences, average weekly distance in the activity wheel tended to be higher in the THR group. Gastrocnemius weight was lower in POL group compared to CON (*p* = 0.038) and *tibialis anterior* was lower in both POL and THR compared to CON (*p* = 0.01). However, these differences were no longer observed when the *gastrocnemius* (*p* = 0.783) and *tibialis anterior* (*p* = 0.821) weights were expressed as a percentage of total bodyweight. (Fig. 3; Appendix S2 of the ESM).

### V̇O_2_max and endurance capacity

**Fig. 4:**
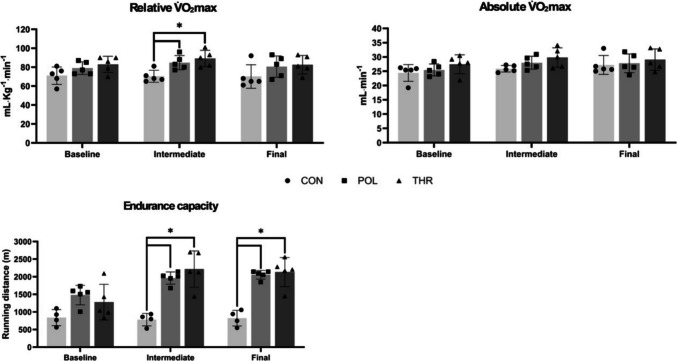
Exercise testing: **A** relative to body weight maximal oxygen consumption (V̇O_2_max; mL^.^Kg^−1.^min^−1^) at baseline, intermediate (four weeks) and final assessment (eight weeks); **B** absolute V̇O_2_max (mL.min^−1^) at baseline, intermediate and final assessment; **C** total distance performed (in meters; m) during the endurance capacity test at baseline, intermediate and final assessment. Data are presented as mean ± standard deviation. CON: Control group: POL: Polarized training group; THR: Threshold training group. Asterisks highlight the existence of statistically significant differences for CON in comparison to POL and THR (*p < 0.05)

There were no differences in baseline relative V̇O_2_max between groups (*p* = 0.101) (Fig. 4A). Two-way ANOVA showed the existence of a group effect, with significant differences between THR and CON (*p* = 0.036), while there we no differences between POL vs CON (*p* = 0.133) or between POL vs THR (*p* = 1.0). Nevertheless, no significant time effect or time*group interaction effect was identified.

Considering that animals significantly increased body mass throughout the protocol, it was relevant to also assed absolute V̇O_2_max (Fig. 4B). Baseline absolute V̇O_2_max was similar between groups (*p* = 0.262). There was also no significant effect of group, time or a time*group interaction effect. Nevertheless, a tendency for an effect of time was identified for both changes in between absolute V̇O_2_max at baseline and 4-weeks of training (*p* = 0.063) and between 4- and 8-weeks of training (*p* = 0.074).

EC was also similar between all groups at baseline, intermediate and final timepoint assessments (Fig. 4C). Nevertheless, a sensitivity analysis was performed, and an extreme outlier was removed from the CON group. After this, two-way ANOVA showed a significant group effect between both training groups and controls (*p* < 0.01), but not between POL and THR (*p* = 1.0). Interestingly, improvements in EC were significant after only 4 weeks of training (*p* < 0.01) while there was no time effect between 4- and 8-weeks of the protocol (*p* = 1.0). There was also no group*time interaction in CON group (*p* = 1.0), while for POL and THR there was a significant group*time interaction between baseline and 4-weeks of training (*p* = 0.12, *p* < 0.01, respectively), but not between 4- and 8- weeks of training (*p* = 0.549, *p* = 0.679, respectively). (Appendix S2 of the ESM).

### High resolution respirometry

**Fig. 5:**
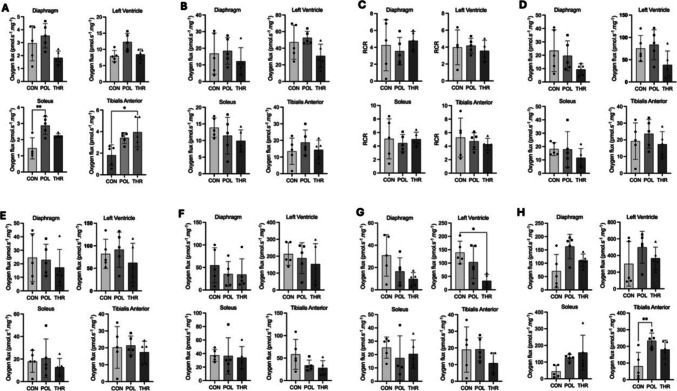
High resolution respirometry data of the diaphragm, left ventricle myocardium, soleus and tibialis anterior muscles of animals from the control (CON), polarized training (POL) and threshold training (THR) following 8 weeks of training. **A** NADH electron transfer-pathway state [(1PM) CI]; **B** OXPHOS capacity [(2D) CI ADP]; **C** respiratory control ratio for ADP [(RCR_ADP_) CI + ADP]; **D** respiratory flow after challenge with cytochrome c (2c) CI + ADP + Cyt c; **E** maximal uncoupled mitochondrial respiration [(3U) CI + ADP + Cyt c + CCCP]; **F** maximal phosphorylating respiration with electrons provided by complexes I and II [(5S) CI and CII + ADP + Cyt c + CCCP]; **G** maximal phosphorylating respiration with electrons provided only by complex II [(7Rot) CII + ADP + Cyt c + CCCP]; **H** maximal phosphorylating respiration with electrons provided only by complex IV [(AsTm) CIV + ADP + Cyt c + CCCP]. Data represent mean and standard deviation. Symbols in each column represent individual data points in each group. Asterisks highlight the existence of statistically significant differences between groups (*p < 0.05, **p < 0.01)

Exercise training increased non-phosphorylating resting state (leak) with electron provision through complex I (Fig. 5A), particularly in the *soleus* and *tibialis anterior* muscles, respectively, after POL and THR training (*p* < 0.005). In the case of the diaphragm and left ventricle, the median value was higher in the POL group, even though differences were not significant. No significant variations in OXPHOS respiration were observed across the groups for any of the tissue samples examined, including the left ventricle (*p* = 0.219), diaphragm (*p* = 0.707), *soleus* (*p* = 0.297) and *tibialis anterior* (*p* = 0.622) (Fig. 5B). Similarly, there were no significant differences in RCR between groups for any of the tissue samples analyzed, namely left ventricle (*p* = 0.850), diaphragm (*p* = 0.653), *soleus* (*p* = 0.827) and *tibialis anterior* (*p* = 0.887) (Fig. 5C). There were no significant differences in respiratory rate after adding cytochrome c between groups, namely at the left ventricle (*p* = 0.145), diaphragm (*p* = 0.209), *soleus* (*p* = 0.470) and *tibialis anterior* (*p* = 0.731) (Fig. 5D). Nevertheless, despite not reaching statistical significance, there was a pronounced downward trend in median values in POL and THR groups compared to CON in both the diaphragm and *soleus* muscles. Furthermore, in the case of mitochondria from the left ventricle, they not only exhibit a greater dispersion of results, but their order of magnitude is also higher when compared with other tissues, especially in CON and POL groups. The rate of maximal uncoupled mitochondrial respiration (CI) in the presence of the uncoupler CCCP (Fig. 5E) showed that there were no significant differences between groups in respiratory rate namely in the left ventricle (*p* = 0.615), diaphragm (*p* = 0.795), *soleus* (*p* = 0.601) and *tibialis anterior* muscles (*p* = 0.895). Regarding oxidative phosphorylation pathway (OXPHOS), our findings indicate that average values for diaphragm, *soleus* and *tibialis anterior* muscles were consistently close to one. Similarly, maximal phosphorylating respiration (CI + II), was not significantly different between groups, namely in the left ventricle (*p* = 0.725), diaphragm (*p* = 0.638), *soleus* (*p* = 0.967) and *tibialis anterior* (*p* = 0.118) (Fig. 5F). Regarding maximal phosphorylating respiration with electron provision exclusively through complex II (Fig. 5G) we observed a significant decrease in oxygen flux in THR group left ventricle (*p* = 0.019) in comparison with CON group. However, no significant differences were observed between POL and CON groups (*p* = 0.561). Furthermore, there were no significant differences between groups in any of the other tissue samples analyzed, including the diaphragm (*p* = 0.118), *soleus* (*p* = 0.638), and *tibialis anterior* (*p* = 0.608). The flow of electrons through Complex IV (CIV/Cytochrome c oxidase, COX) (Fig. 5H), except for the result obtained in the *tibialis anterior*, in which the POL group was statistically different from CON (*p* = 0.007), showed no significant differences in any of the other tissue samples analyzed, including the diaphragm (*p* = 0.091), left ventricle (*p* = 0.331), and *soleus* (*p* = 0.091). Notably, although not achieving statistical significance, there was a pronounced tendency towards increased respiration, contrasting with what was observed for CI and CII.

### Diaphragm histological analysis

**Fig. 6:**
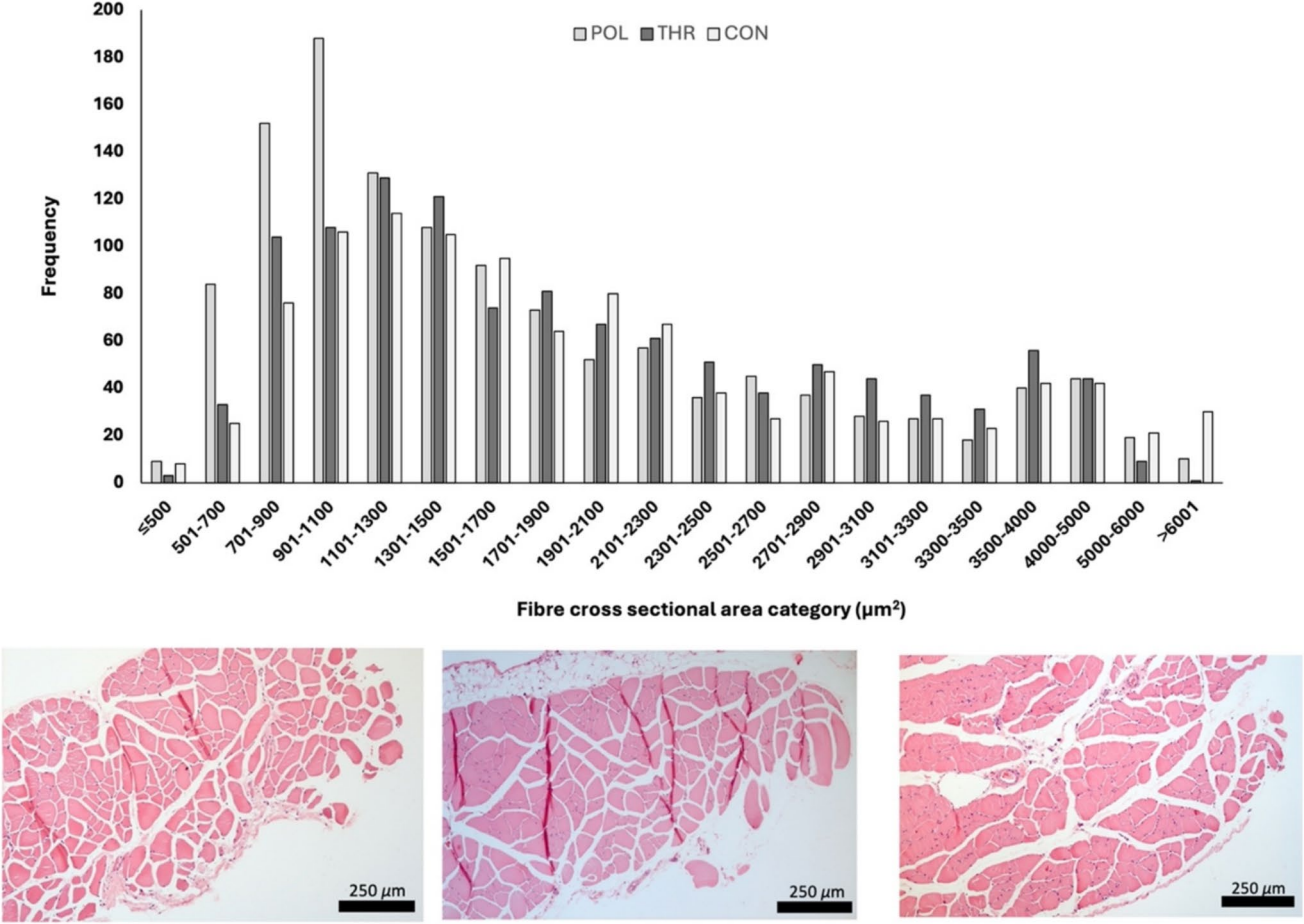
Diaphragm muscle fiber cross-sectional area (µm^2^). X axis represents the cross-sectional area range (µm^2^) and Y axis represents the number of diaphragm muscle fibers. CON: Control group; POL: Polarized training group; THR: Threshold training group. Asterisks highlight the existence of statistically significant differences between groups (*p < 0.05)

Approximately 250 muscle fibers of the diaphragm of each animal were analyzed. There were no significant differences between groups in diaphragm muscle fiber cross-sectional area. Additionally, the distribution frequency of fibers cross-sectional area between groups was also identical, except for fibers in the 901–1100 µm^2^ range, which were significantly more prevalent in POL (*p* = 0.041). In all groups, the highest number of muscle fibers was within the range of 700 and 1700 *µ*m^2^ (Fig. 6; Appendix S2 of the ESM).

### Citrate synthase (CS) activity

**Fig. 7:**
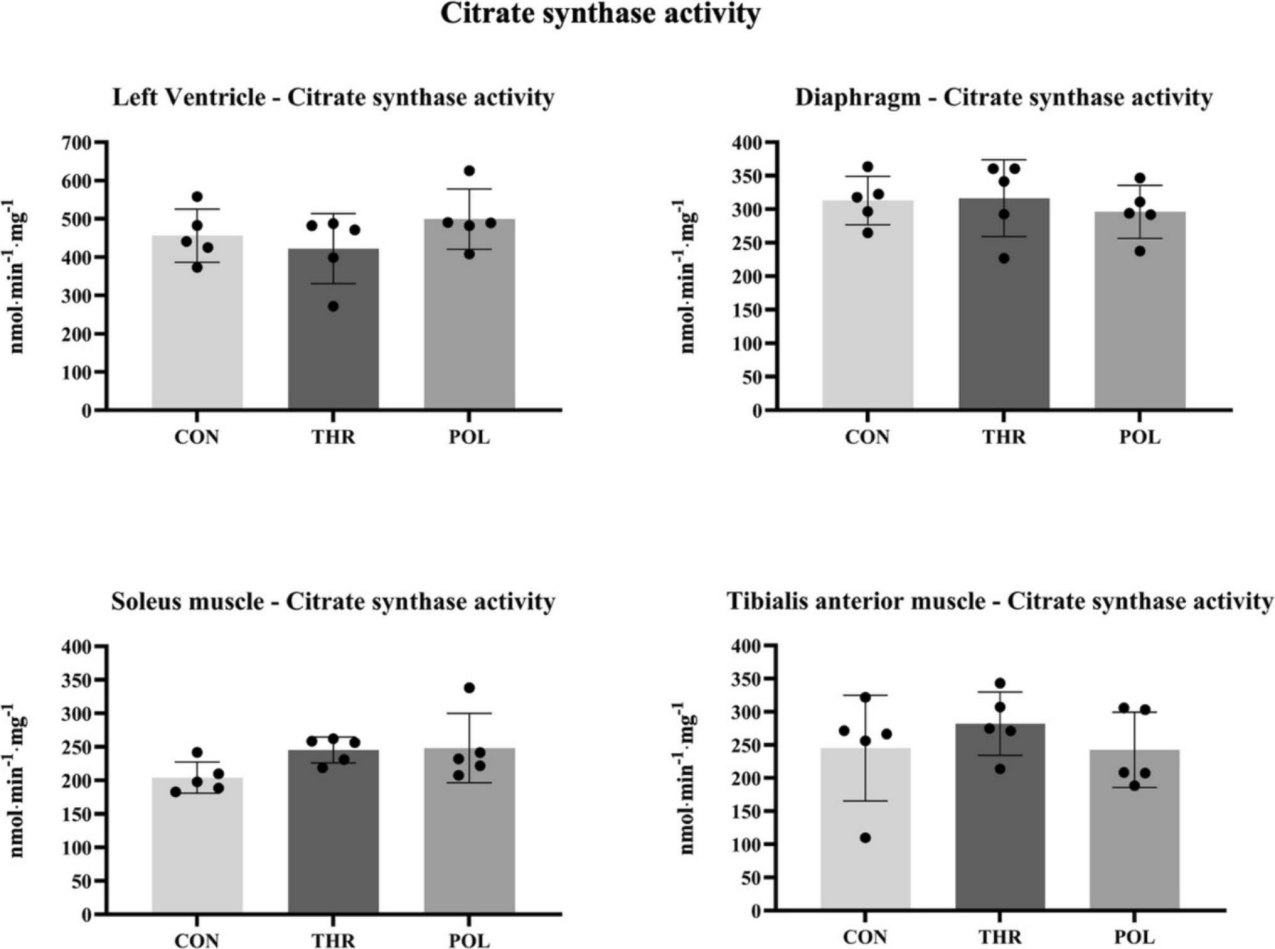
Citrate synthase (CS) activity in the several tissues analyzed. Values (mean ± SD) are expressed in nmol.min^−1^.mg^−1^ of *n* = 5 for CS activity. Control group: POL: Polarized training group; THR: Threshold training group

Citrate synthase (CS) activity was determined in tissue samples of the left ventricle myocardium, diaphragm, *soleus*, and *tibialis anterior* muscles collected at the end of the training protocol. There were no significant differences in CS activity between groups regarding all tissue samples analyzed (Fig. 7; Appendix S2 of the ESM).

### Mitochondrial biogenesis and dynamics biomarkers

**Fig. 8:**
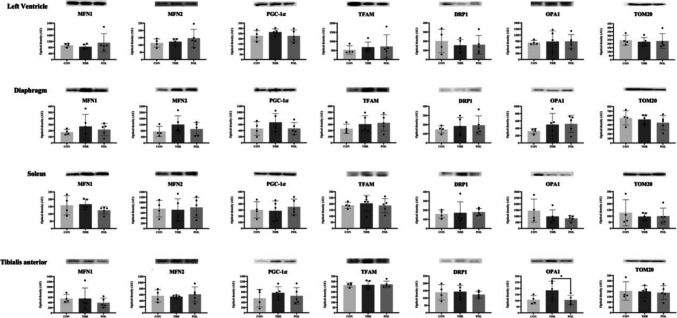
Mitochondrial dynamics markers MFN1, MFN2, PGC-1α, TFAM, DRP1, OPA1 and TOM20. Effects of training in MFN1 (Mitofusin 1), MFN2 (Mitofusin 2), PGC-1α (Peroxisome proliferator-activated receptor gamma coactivator-1 alpha), TFAM (Mitochondrial transcription factor A), DRP1 (dynamin-related protein 1), OPA1 (optic atrophy protein 1) and TOM20 (translocase of outer mitochondrial membrane 20) expression measured by Western Blot. Above each graph there is a representative image of the bands obtained. Samples were loaded into the gel one per group side-by-side. Values (mean ± SD) are expressed in arbitrary units of optical density (OD) of *n* = 5, relativized to Ponceaus S. CON: Control group: POL: Polarized training group; THR: Threshold training group. Asterisks highlight the existence of statistically significant differences between groups (*p < 0.05)

There were no significant differences between groups regarding the expression of any of the mitochondrial biogenesis (PGC-1α, TFAM) and dynamics biomarkers assessed (MFN1, MFN2, DRP1, OPA1 and TOM20) in all samples analyzed (Fig. 8; Appendix S2 of the ESM). The only exception was regarding OPA1 expression in tibialis anterior which was higher in THR compared to POL (*p* = 0.049).

## Discussion

Our results suggest that both THR and POL are equally effective improving V̇O_2_max and EC at the extent. Furthermore, there was no clear evidence of superiority of either TID regarding mitochondrial oxidative phosphorylation, muscle mass, cardiac or diaphragm structural adaptations that would suggest any mechanistic superiority of any of the two TIDs.

After four weeks of training, V̇O_2_max of both POL and THR were significantly higher compared to CON. However, there were no differences between POL and THR. This supports the notion that previously untrained subjects are equally sensitive to various TIDs, improving similarly at the beginning of a training program, irrespectively of the training stimulus [25]. Nevertheless, at the end of the protocol, there were no differences between groups regarding relative or absolute V̇O_2_max. Our results do not align with Inoue et al., [45] which showed that rats training above the lactate threshold for 6 weeks showed a 12.7% improvement in V̇O_2_max compared to rats training below the lactate threshold or the control group. Absence of differences between trained and control animals could be explained by the fact that some animals in the CON group were naturally very active on the running wheel. Given that running pattern in the wheel is mainly characterized by intermittent bouts of high intensity running [4], this may have contributed to their high V̇O_2_max and, consequently lack of differences between groups. Also, absence of differences between POL and THR is in accordance with previous findings showing absence of V̇O_2_max differences between recreational endurance [24] and ultra-endurance runners [68] performing eight or 12 weeks of POL *vs* THR. Nonetheless, Stöggl et al., [87] concluded that POL was superior to THR (11.7% *vs* −4.1%) on highly trained endurance athletes (V̇O_2_max: 62.6 ± 7.1 mL_•_Kg^−1^_•_min^−1^) after nine weeks of training. This suggests that in previously trained athletes POL can induce superior adaptations compared to untrained subjects.

V̇O_2_ max is a key determinant of endurance performance but is not its major surrogate. EC was therefore assessed and was significantly higher in POL and THR compared to CON at intermediate and final assessments. However, there were no differences between POL and THR at both timepoints. These results differ from several previous studies in athletes suggesting POL superiority in time-to-exhaustion [64, 68, 87]. This superiority seems to result specifically from adaptations triggered by high intensity exercise, favoring mitochondrial biogenesis [3, 29] and higher recruitment of fast-twitch muscle fibers, conferring higher fatigue resistance at vigorous intensities, extending time to exhaustion [72, 96]. These results might stem from the high daily activity in the running wheel by POL and THR, which could have mitigated eventual differences induced by each specific training protocol. For instance, despite lack of wheel running differences, there was a higher number of THR (*n* = 5) animals running > 10 km/week compared to POL (*n* = 2), which might have compensated their lower running protocol intensity.

Respiratory muscle function is a critical aspect in endurance performance [81]. During high intensity exercise, breathing frequency increases exponentially, causing diaphragm fatigue and inability to sustain exercise intensity. Considering that low-to-moderate-intensity exercise is associated with decreases in diaphragm muscle fiber CSA [74], we hypothesized that diaphragm function would improve with high intensity training in POL group. Contrarily to our initial hypothesis, there were no differences in diaphragm mass, fiber CSA or in any of the remaining bioenergetic variables assessed.

Endurance training enhances mitochondrial number [31], structure and function [52] namely its oxidative capacity and efficiency [15, 41, 63, 82, 86, 89]. Our results show that POL and THR exhibited comparable bioenergetic adaptations, evidenced by similar OXPHOS capacity, CS activity, and mitochondrial biogenesis (PGC-1, TFAM) and dynamics (MFN1, MFN2, DRP1, OPA1, TOM20) markers in the multiple tissues analyzed. This is in agreement with our functional results, showing that different TID’s resulted in similar endurance performance.

It is also important to highlight that some training adaptations are slow and, consequently, it is conceivable that protocol duration could have been insufficient to trigger significant differences between groups. Nevertheless, evidence in humans suggests that mitochondrial respiration improvements could be achieved with ≤ 10 weeks of training in protocols combining moderate intensity continuous training (MICT) and HIIT [36]. For instance, 10 weeks of MICT and HIIT resulted in 38% enhancement in *vastus lateralis* OXPHOS capacity [71]. Four days/week, six-weeks MICT and Sprint Interval Training (SIT) also resulted in 42% increases in vastus lateralis OXPHOS capacity [92].

Considering the importance of PGC-1α for mitochondrial biogenesis, we also investigated its expression on several tissues [98]. Again, our findings showed no differences in PGC-1α expression between groups, suggesting that mitochondrial biogenesis was similarly stimulated irrespectively of training TID. Regarding TFAM expression, there are conflicting findings about the effect of endurance training [35] with studies showing unchanged [34, 38] or increased [5, 33, 55] TFAM expression after different exercise training protocols. Granata et al., [35] suggests TFAM expression is mainly affected by training volume and not intensity. This is in line with our findings since training volume was equalized between POL and THR groups to ensure isocaloric training conditions and, despite the high heterogeneity, there were no significant differences in daily physical activity between groups. Mitochondrial fusion is enhanced by acute [42] and chronic endurance exercise [2]. In agreement with our previous results, there were also no differences in MFN1, MFN2 DRP1, OPA1 and TOM20 expression between POL and THR groups with the exception of OPA1 expression in tibialis anterior.

CS is a key enzyme in mitochondrial function and frequently assessed as a surrogate for endurance training adaptations [84]. Nevertheless, different alterations have been documented in CS activity in response to endurance training, with most studies documenting a rise (MICT [1, 6, 21, 30, 60, 62, 76], HIIT [33, 38, 47, 69, 70, 91] and SIT [30, 39, 95, 99]) while some have shown no changes (MICT [12, 34, 37, 59], HIIT [34], SIT [22, 53, 58]). A similar pattern appears to be observed with animal models. Leandro et al., [51] found an increase in CS activity of the *soleus* muscle from trained rats after a 8-week moderate endurance training protocol based on V̇O_2_max. Conversely, in the study of Carvalho et al., [18], no differences in CS or OXPHOS activity were observed between low, moderate, and high intensity aerobic training with equalized loads in Wistar rats. Burgomaster et al., [6] also showed that CS activity increased similarly after both six weeks of SIT or traditional endurance training at moderate intensity. These findings suggest that adaptations in CS activity were unaffected by training intensity and respond equally to similar training volumes. Despite this, Neal et al., [64] in a study in trained cyclists that performed a six-week POL protocol, it was observed a greater CS activity in POL compared to THR athletes even though training volume was (theoretically) controlled and equalized according to the TRIMP approach [57].

Regarding high-resolution respirometry, our results highlight that exercise training influenced specific aspects of mitochondrial function. The increased non-phosphorylating resting state (leak) in *soleus* and *tibialis anterior* muscles after POL and THR training may indicate enhanced mitochondrial adaptability through Complex I. However, the lack of significant changes in OXPHOS capacity across tissues suggests that maximal ATP production capacity was not affected by the different training protocols. The consistent RCR_ADP_ across groups reinforces this observation, indicating maintained oxidative phosphorylation efficiency. It is also conceivable that the time of recovery after the V̇O_2_max and time to exhaustion protocols might have been insufficient to allow complete muscle recovery, particularly in THR and POL groups in which higher external loads were achieved. In line with this notion, Trewin et al., [94] shows a rise in uncoupled leak respiration subsequent to HIIT and sprint interval training (SIT) while Peden et al., [67] showed similar findings following a time-to-exhaustion test at 92% maximal aerobic power. Consequently, this scenario is believed to have led to an elevation in the leak state, indicative of compromised mitochondrial function, characterized by increased proton leak and diminished ATP resynthesis efficiency. Nevertheless, with respect to cytochrome c-stimulated respiration, the absence of significant changes suggests that the mitochondrial outer membrane integrity was preserved across exercise protocols, with the exception of the left ventricle. Also, variability in the left ventricle results may stem from mechanical homogenization challenges, potentially impacting membrane integrity. It is important to note that the mitochondria in cardiac muscle displayed a higher degree of outer membrane damage during the homogenization process using the PBI-Shredder O2K, as expected due to their lower resistance to mechanical stress. However, this particularity has only a minimal effect on the results interpretation since cytochrome c supplementation during high-resolution respirometry restores electron transport, ensuring the maintenance of electron transport through the inner mitochondrial membrane. Regarding maximal uncoupled respiration (CI) and CI + II phosphorylation, although this parameter did not differ significantly between groups, the trends toward lower oxygen flux in trained groups warrants suggests the exercise-induced shifts in electron transport chain efficiency.

Interestingly, these results are in opposition to a recently published meta-analysis suggesting the superiority of POL regarding V̇O_2_max improvements [83]. We consider that this contradiction strengthens the notion that evidence supporting POL superiority in studies with athletes is likely based on experiments lacking a thorough control of key training variables [7] such as intensity, as well as possible lack of isocaloric comparisons between training protocols. Another potential explanation for the lack of significant differences between POL, THR, and CON may be due to the use of running wheels in the cages, leading to high levels of voluntary physical activity in CON group. This spontaneous activity could have masked potential differences in V̇O_2_max and biochemical markers, since the free-wheel running model has been shown to mimic the exercise pattern seen in HIIT [65] induces several typical endurance training adaptations including enhanced mitochondrial mass, improved bioenergetics and increases in V̇O_2_max [4, 73].

A key limitation of this study is the lack of electron microscopy to assess mitochondrial morphology and cristae density, both of which are essential for understanding mitochondrial function. Tissues with similar mitochondrial contents can still present substantial differences in mitochondrial morphology and cristae density potentially affecting cellular bioenergetics [11, 14]. While we measured molecular markers of mitochondrial dynamics, biogenesis, and respiratory function, combining these with structural data would provide an improved picture of mitochondrial health and functionality. Another important limitation of our study is that it was designed to be able to detect only large magnitude differences between groups. As a result, the reduced sensitivity of the experimental design may have limited the ability to identify statistically significant differences in some variables, especially regarding mitochondrial bioenergetics results [46].

The main strengths of this study are the rigorous control of training variables and comprehensive bioenergetic analysis performed encompassing several tissues. By scrutinizing parameters such as mitochondrial OXPHOS and CS activity, and the expression of key regulatory proteins across multiple tissues, this research provides valuable insights into the physiological adaptations underlying different TIDs. Furthermore, the meticulous prescription and monitoring of training loads in a standardized and rigorous manner contributed to the methodological robustness. However, this study also has limitations. The relatively short duration of the protocol raises the possibility that different results could have been obtained with longer interventions. Additionally, while efforts were made to maintain an ecologically valid and controlled environment during training and assessments, the potential impact of the animals' daily physical activity on the observed outcomes cannot be discounted. The high variability of daily physical activity may have contributed to our findings heterogeneity, potentially obscuring the specific effects of each TID. Thus, future studies should compromise the ecological validity of the model by removing access to the running wheel at the expense of increasing the control over the animals daily physical activity. Our results suggest that there is no evidence supporting the superiority of POL in the improvement of endurance performance nor does it seem to be evidence of a higher degree of cardiac or muscle bioenergetic adaptations as a result of POL training. Nevertheless, in our experimental study we did not investigate hematological adaptations to training. Considering the importance of the blood O_2_ transport capacity for endurance performance, future studies should address this aspect to be able to determine if, in fact, there are no major differences in physiological adaptations to POL and THR.

## Conclusion

There were no significant differences in V̇O_2_max and endurance capacity between POL and THR at the end of eight weeks of endurance training. Diaphragm muscle fiber cross sectional area was identical between groups. POL and THR groups displayed similar bioenergetic adaptations regarding mitochondrial OXPHOS capacity, CS activity and PGC-1α, TFAM, MFN1, MFN2, DRP1, OPA1 and TOM20 expression at the left ventricle myocardium, diaphragm, *soleus* and *tibialis anterior* muscles. These findings have practical implications for coaches and exercise scientists, emphasizing the potential interchangeability of POL and THR training approaches in achieving similar physiological outcomes. Nevertheless, these conclusions should be interpreted with caution, considering the reduced sample size and statistical power and the possibility that some potential differences may have not reached statistical significance due to the small sample size.

## Supplementary Information

Below is the link to the electronic supplementary material.

Supplementary file1 (DOCX 230 KB)

Supplementary file2 (DOCX 54 KB)

Supplementary file3 (DOCX 15574 KB)

## Data Availability

No datasets were generated or analysed during the current study.

## Abbreviations

ADP: Adenosine diphosphate;
Ama: Antimycin A;
AMPK: 5' AMP-activated protein kinase;
As: Ascorbate;
ATP: Adenosine trisphosphate;
Azd: Sodium azide;
Cyt. c: Cytochrome c;
CCCP: Uncoupler CCCP—Carbonyl Cyanide-m-Chlorophenylhydrazone;
CON: Control group;
CS: Citrate synthase;
D: Adenosine diphosphate (ADP) + Mg2 + ;
DRP1: Dynamin-related protein 1
DMSO: Dimethyl sulfoxide;
EC: Endurance capacity;
EtOH: Ethanol;
G: Glutamate;
Gp: Glycerophosphate;
H_2_O: Water;
HITT: High intensity interval training;
M: Malate;
MFN1: Mitofusin 1;
MFN2: Mitofusin 2;
MICT: Moderate intensity continuous training;
NAD +: Nicotinamide adenine dinucleotide;
NADH: Nicotinamide adenine dinucleotide + hydrogen;
O: Octanoylcarnitine;
OPA1: Optic atrophy protein 1
OXPHOS: Oxidative phosphorylation;
P: Pyruvate (fresh preparation);
PGC-1α: Peroxisome proliferator-activated receptor gamma coactivator 1-alpha;
POL: Polarized training;
RCR: Respiratory control ratio for ADP;
RER: Respiratory exchange ratio;
Rot: Rotenone (inhibitor);
S: Succinate;
SIT: Sprint interval training;
TFAM: Mitochondrial transcription factor A;
THR: Threshold training;
TID: Training intensity distribution;
Tm, TMPD: N,N,N',N'-Tetramethyl-phenylenediamine dihydrochloride;
TOM20: Translocase of outer mitochondrial membrane 20
V̇O_2_max: Maximal oxygen uptake
