# Supplementary material for: Polarized or threshold training: is there a superior training intensity distribution to improve V̇O2max, endurance capacity and mitochondrial function? A study in Wistar Rat models
Source: J Physiol Biochem. 2025 Apr 2;81(2):329–46. doi: 10.1007/s13105-025-01079-6 (PMC12279575; doi:10.1007/s13105-025-01079-6)
Supplement: Supplementary file 1 — Supplementary file1 (DOCX 230 KB) [file 13105_2025_1079_MOESM1_ESM.docx]

**Online Resource S1**

**Title: Polarized or threshold training: is there a superior training intensity distribution to improve VO_2_max, endurance capacity and mitochondrial function? A study in Wistar Rat models**

**Journal:** Journal of Physiology and Biochemistry

**Authors:** *Oliveira, Pedro^1,,2,3^; Anjos, Miguel^1, 2^; Flores, Ariane^1,2^;* Peixoto, Francisco^4^; Padrão, Ana Isabel^1,2^; *Fonseca, Hélder^1,2^*

*^1^Research Centre in Physical Activity, Health and Leisure (CIAFEL); Faculty of Sport of University of Porto (FADE-UP), Porto, Portugal*

*^2^Laboratory for Integrative and Translational Research in Population Health (ITR), Porto, Portugal*

^3^*Nucleus of Research in Human Motricity Sciences, Universidad Adventista de Chile, Chillán 3780000, Chile*

*^4^Vila Real Chemistry Center (CQVR), Biology and Environment Department, University of Trás-os-Montes and Alto Douro, 5000-801 Vila Real, Portugal*

**Corresponding author:** Pedro Oliveira ([up201807240@fade.up.pt](mailto:up201807240@fade.up.pt))

**Electronic Supplementary Material Appendix S1**

**Table S1** - O2k manual titrations of SUIT-001 O2 mt D001 protocol for skeletal and cardiac muscle tissues in O2k-sVModule - Mitochondrial Physiology Network 09.12(20):1-4 (2020).

**
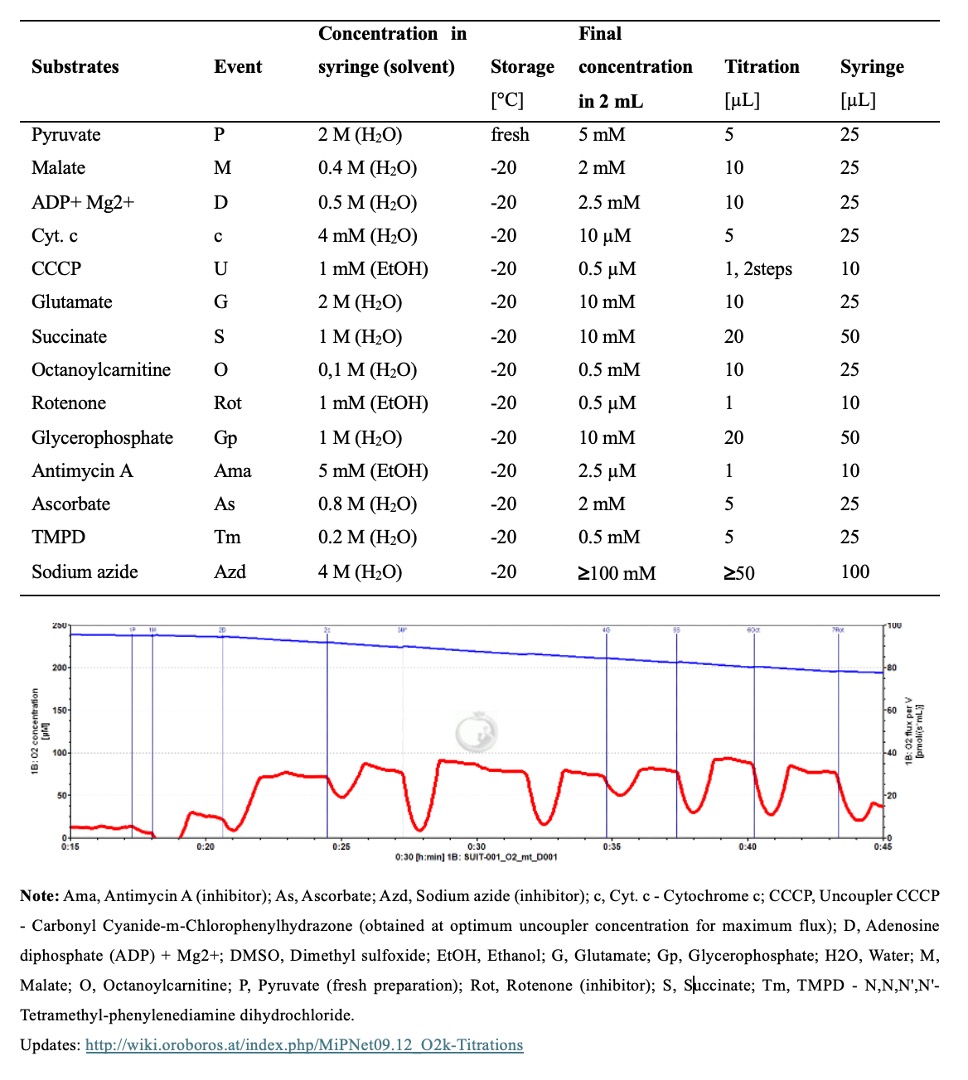
**

**Note:** Ama, Antimycin A (inhibitor); As, Ascorbate; Azd, Sodium azide (inhibitor); c, Cyt. c - Cytochrome c; CCCP, Uncoupler CCCP - Carbonyl Cyanide-m-Chlorophenylhydrazone (obtained at optimum uncoupler concentration for maximum flux); D, Adenosine diphosphate (ADP) + Mg2+; DMSO, Dimethyl sulfoxide; EtOH, Ethanol; G, Glutamate; Gp, Glycerophosphate; H2O, Water; M, Malate; O, Octanoylcarnitine; P, Pyruvate (fresh preparation); Rot, Rotenone (inhibitor); S, Succinate; Tm, TMPD - N,N,N',N'-Tetramethyl-phenylenediamine dihydrochloride.

Updates: <http://wiki.oroboros.at/index.php/MiPNet09.12_O2k-Titrations>
