# Supplementary material for: Polarized or threshold training: is there a superior training intensity distribution to improve V̇O2max, endurance capacity and mitochondrial function? A study in Wistar Rat models
Source: J Physiol Biochem. 2025 Apr 2;81(2):329–46. doi: 10.1007/s13105-025-01079-6 (PMC12279575; doi:10.1007/s13105-025-01079-6)
Supplement: Supplementary file 2 — Supplementary file2 (DOCX 54 KB) [file 13105_2025_1079_MOESM2_ESM.docx]

**Online Resource S2**

**Title: Polarized or threshold training: is there a superior training intensity distribution to improve VO_2_max. endurance capacity and mitochondrial function? A study in Wistar Rat models**

**Journal:** Journal of Physiology and Biochemistry

**Authors:** *Oliveira. Pedro^1.2,3^; Anjos. Miguel^1. 2^; Flores. Ariane^1.2^;* Peixoto. Francisco^4^; Padrão. Ana Isabel^1.2^; *Fonseca. Hélder^1.2^*

*^1^Research Centre in Physical Activity. Health and Leisure (CIAFEL); Faculty of Sport of University of Porto (FADE-UP). Porto. Portugal*

*^2^Laboratory for Integrative and Translational Research in Population Health (ITR). Porto. Portugal*

^3^*Nucleus of Research in Human Motricity Sciences, Universidad Adventista de Chile, Chillán 3780000, Chile*

*^4^Vila Real Chemistry Center (CQVR). Biology and Environment Department. University of Trás-os-Montes and Alto Douro. 5000-801 Vila Real. Portugal*

**Corresponding author:** Pedro Oliveira ([up201807240@fade.up.pt](mailto:up201807240@fade.up.pt))

**Electronic Supplementary Material Appendix S2**

**Table S1 –** Sample characterization. functional. structural and bioenergetic variables

| **Variable** | **CON** | | **POL** | | **THR** | | **p** |
| --- | --- | --- | --- | --- | --- | --- | --- |
|  | **mean** | **SD** | **mean** | **SD** | **mean** | **SD** |  |
| **Sample characterization** |  |  |  |  |  |  |  |
| Baseline bodyweight (g) | 352 | 40 | 325 | 26 | 331 | 21 | 0.362 |
| 2^nd^ week bodyweight (g) | 342 | 30 | 321 | 17 | 326 | 17 | 0.318 |
| 3^rd^ week bodyweight (g) | 345 | 21 | 323 | 17 | 330 | 14 | 0.178 |
| 4^th^ week bodyweight (g) | 354 | 22 | 333 | 15 | 332 | 17 | 0.142 |
| 5^th^ week bodyweight (g) | 356 | 26 | 331 | 16 | 334 | 16 | 0.128 |
| 6^th^ week bodyweight (g) | 361 | 29 | 326 ^a^ | 19 | 333 | 11 | **0.048** |
| 7^th^ week bodyweight (g) | 369 | 30 | 329 ^a^ | 21 | 341 | 13 | **0.040** |
| 8^th^ week bodyweight (g) | 369 | 28 | 331 ^a^ | 23 | 341 | 15 | **0.049** |
| 9^th^ week bodyweight (g) | 377 | 28 | 339 | 25 | 348 | 13 | 0.058 |
| 10^th^ week bodyweight (g) | 383 | 27 | 337 ^a^ | 22 | 351 | 14 | **0.017** |
| 11^st^ week bodyweight (g) | 386 | 23 | 340 ^a^ | 19 | 355 ^b^ | 9 | **0.005** |
| 12^nd^ week bodyweight (g) | 389 | 24 | 344 ^a^ | 18 | 353 ^b^ | 9 | **0.005** |
| 13^rd^ week bodyweight (g) | 390 | 24 | 348 ^a^ | 20 | 352 ^b^ | 11 | **0.008** |
| 14^th^ week bodyweight (g) | 391 | 25 | 351 ^a^ | 20 | 359 | 11 | **0.015** |
| Final bodyweight (g) | 399 | 28 | 359^a^ | 21 | 365 | 10 | **0.020** |
| Mass variation (g) | 47.2 | 16.6 | 33.8 | 14.1 | 41.0 | 11.2 | 0.370 |
| Heart mass (g) | 0.986 | 0.080 | 0.948 | 0.061 | 1.000 | 0.032 | 0.401 |
| Diaphragm mass (g) | 1.003 | 0.057 | 0.952 | 0.065 | 1.014 | 0.066 | 0.304 |
| *Soleus* mass (g) | 0.324 | 0.039 | 0.278 | 0.033 | 0.276 | 0.029 | 0.078 |
| *Gastrocnemius* mass (g) | 5.07 | 0.34 | 4.61^a^ | 0.14 | 4.74 | 0.23 | **0.033** |
| *Tibialis anterior* mass (g) | 1.640 | 0.057 | 1.444^a^ | 0.434 | 1.498^a^ | 0.073 | **0.001** |
| *Triceps brachii* mass (g) | 3.41 | 0.37 | 3.44 | 0.14 | 3.51 | 0.12 | 0.825 |
| Average food intake (g.week^-1^) | 142.5 | 17.1 | 136.4 | 10.4 | 152.2 | 14.1 | 0.245 |
| Average voluntary PA (km.week^-1^) | 11.1 | 17.9 | 11.1 | 16.4 | 24.0 | 25.3 | 0.181 |
| **Exercise testing** |  |  |  |  |  |  |  |
| Baseline V̇O_2_max (ml^.^kg^-1.^min^-1^) | 71.0 | 9.1 | 79.0 | 6.6 | 83.0 | 8.5 | 0.101 |
| Intermediate V̇O_2_max (ml^.^kg^-1.^min^-1^) | 70.4 | 6.3 | 84.8^a^ | 7.4 | 89.2^b^ | 8.5 | **0.005** |
| Final V̇O_2_max (ml^.^kg^-1.^min^-1^) | 70.2 | 12.4 | 80.6 | 11.3 | 82.6 | 9.7 | 0.213 |
| Baseline Endurance Capacity (m) | 906 | 247 | 1480 | 276 | 1278 | 507 | 0.076 |
| Intermediate Endurance Capacity (m) | 782 | 178 | 1963 ^a^ | 173 | 2218 ^b^ | 516 | **0.001** |
| Final Endurance Capacity (m) | 824 | 223 | 2057 ^a^ | 122 | 2134 ^b^ | 413 | **0.001** |
| **Structural assessments** |  |  |  |  |  |  |  |
| Diaphragm fiber CSA (µm^2^) | 2191 | 368 | 1790 | 466 | 1951 | 412 | 0.343 |
| CSA <500 (µm^2^) frequency | 1.6 | 2.1 | 1.8 | 2.2 | 0.6 | 0.5 | 0.531 |
| CSA 501-700 (µm^2^) frequency | 5.0 | 6.2 | 16.8 | 12.5 | 6.6 | 3.6 | 0.90 |
| CSA 701-900 (µm^2^) frequency | 15.2 | 12.0 | 30.4 | 16.2 | 20.8 | 8.5 | 0.199 |
| CSA 901-1100 (µm^2^) frequency | 21.2 | 11.8 | 37.6 ^a^ | 7.5 | 21.6 | 10.7 | **0.41** |
| CSA 1101-1300 (µm^2^) frequency | 22.8 | 7.5 | 26.2 | 6.3 | 25.8 | 16.0 | 0.865 |
| CSA 1301-1500 (µm^2^) frequency | 21.0 | 9.4 | 21.6 | 6.1 | 24.2 | 9.4 | 0.819 |
| CSA 1501-1700 (µm^2^) frequency | 19.0 | 7.7 | 18.4 | 3.2 | 14.8 | 6.3 | 0.510 |
| CSA 1701-1900 (µm^2^) frequency | 12.8 | 7.3 | 14.6 | 4.4 | 16.2 | 4.8 | 0.646 |
| CSA 1901-2100 (µm^2^) frequency | 16.0 | 9.9 | 10.4 | 4.4 | 13.4 | 7.1 | 0.516 |
| CSA 2101-2300 (µm^2^) frequency | 13.4 | 7.2 | 11.4 | 2.6 | 12.2 | 2.4 | 0.794 |
| CSA 2301-2500 (µm^2^) frequency | 7.6 | 5.3 | 7.2 | 3.0 | 10.2 | 3.7 | 0.481 |
| CSA 2501-2700 (µm^2^) frequency | 5.4 | 3.9 | 9.0 | 3.4 | 7.6 | 2.3 | 0.254 |
| CSA 2701-2900 (µm^2^) frequency | 9.4 | 4.0 | 7.4 | 2.5 | 10.0 | 4.9 | 0.566 |
| CSA 2901-3100 (µm^2^) frequency | 5.2 | 4.1 | 5.6 | 1.9 | 8.8 | 5.9 | 0.377 |
| CSA 3101-3300 (µm^2^) frequency | 5.4 | 2.4 | 5.4 | 3.8 | 7.4 | 5.9 | 0.704 |
| CSA 3301-3500 (µm^2^) frequency | 4.6 | 3.0 | 3.6 | 1.7 | 6.2 | 5.4 | 0.548 |
| CSA 3501-4000 (µm^2^) frequency | 8.4 | 3.6 | 8.0 | 7.3 | 11.2 | 10.5 | 0.777 |
| CSA 4001-5000 (µm^2^) frequency | 8.4 | 3.4 | 8.8 | 13.0 | 9.0 | 9.7 | 0.995 |
| CSA 5001-6001 (µm^2^) frequency | 4.8 | 3.3 | 3.8 | 6.3 | 1.8 | 2.5 | 0.555 |
| CSA >60001 (µm^2^) frequency | 6.0 | 7.9 | 0.2 | 0.4 | 2.0 | 1.6 | 1.72 |
| **Bioenergetics** |  |  |  |  |  |  |  |
| **CS activity (nmol.min^-1^mg^-1^)** |  |  |  |  |  |  |  |
| Left ventricle | 456 | 69 | 499 | 79 | 422 | 92 | 0.348 |
| Diaphragm | 313 | 36 | 296 | 39 | 316 | 57 | 0.753 |
| *Soleus* | 204 | 23 | 248 | 52 | 245 | 19 | 0.121 |
| *Tibialis anterior* | 245 | 80 | 243 | 57 | 282 | 48 | 0.558 |
| **Mitochondrial dynamics markers** |  |  |  |  |  |  |  |
| **MFN1 (OD)** |  |  |  |  |  |  |  |
| Left ventricle | 944 | 123 | 1110 | 602 | 844 | 169 | 0.557 |
| Diaphragm | 178 | 45 | 218 | 99 | 268 | 199 | 0.621 |
| *Soleus* | 159 | 66 | 124 | 25 | 165 | 34 | 0.313 |
| *Tibialis anterior* | 550 | 159 | 388 | 181 | 545 | 412 | 0.613 |
| **MFN2 (OD)** |  |  |  |  |  |  |  |
| Left ventricle | 114 | 28 | 147 | 61 | 122 | 23 | 0.518 |
| Diaphragm | 752 | 334 | 907 | 419 | 1197 | 580 | 0.373 |
| *Soleus* | 760 | 315 | 806 | 378 | 713 | 426 | 0.929 |
| *Tibialis anterior* | 560 | 204 | 612 | 244 | 534 | 52 | 0.789 |
| **DRP1 (OD)** |  |  |  |  |  |  |  |
| Left ventricle | 201 | 128 | 154 | 65 | 161 | 102 | 0.607 |
| Diaphragm | 148 | 41 | 193 | 102 | 183 | 94 | 0.732 |
| *Soleus* | 157 | 44 | 178 | 38 | 170 | 121 | 0.227 |
| *Tibialis anterior* | 139 | 52 | 123 | 23 | 143 | 44 | 0.327 |
| **OPA1 (OD)** |  |  |  |  |  |  |  |
| Left ventricle | 835 | 104 | 889 | 333 | 876 | 527 | 0.665 |
| Diaphragm | 323 | 70 | 519 | 235 | 498 | 304 | 0.399 |
| *Soleus* | 1452 | 943 | 818 | 220 | 972 | 546 | 0.515 |
| *Tibialis anterior* | 107 | 34 | 106 | 38 | 183 ^c^ | 74 | **0.035** |
| **TOM20 (OD)** |  |  |  |  |  |  |  |
| Left ventricle | 243 | 62 | 223 | 54 | 233 | 92 | 0.920 |
| Diaphragm | 552 | 157 | 515 | 102 | 445 | 157 | 0.882 |
| *Soleus* | 128 | 106 | 96 | 30 | 100 | 65 | 0.927 |
| *Tibialis anterior* | 204 | 87 | 199 | 55 | 188 | 66 | 0.905 |
| **Mitochondrial biogenesis** |  |  |  |  |  |  |  |
| **PGC-1𝛼 (OD)** |  |  |  |  |  |  |  |
| Left ventricle | 228 | 51 | 227 | 57 | 263 | 30 | 0.423 |
| Diaphragm | 466 | 225 | 467 | 194 | 667 | 290 | 0.363 |
| *Soleus* | 188 | 26 | 187 | 57 | 204 | 65 | 0.858 |
| *Tibialis anterior* | 557 | 334 | 648 | 316 | 762 | 239 | 0.595 |
| **TFAM (OD)** |  |  |  |  |  |  |  |
| Left ventricle | 890 | 651 | 630 | 315 | 474 | 216 | 0.353 |
| Diaphragm | 230 | 75 | 326 | 134 | 303 | 140 | 0.515 |
| *Soleus* | 303 | 132 | 352 | 127 | 286 | 161 | 0.757 |
| *Tibialis anterior* | 265 | 20 | 272 | 31 | 267 | 39 | 0.950 |

| **High-resolution respirometry** |  |  |  |  |  |  |  |
| --- | --- | --- | --- | --- | --- | --- | --- |
| **Complex I (1PM - CI)** |  |  |  |  |  |  |  |
| Left ventricle | 7.994 | 1.739 | 12.284 ^a^ | 2.697 | 8.433 | 1.673 | **0.012** |
| Diaphragm | 2.946 | 1.355 | 3.531 | 0.940 | 1.819 | 0.530 | 0.053 |
| *Soleus* | 1.467 | 0.655 | 2.876 ^a^ | 0.549 | 2.247 | 0.120 | **0.004** |
| *Tibialis anterior* | 1.815 | 0.963 | 3.414 | 0.507 | 3.949 ^b^ | 1.376 | **0.016** |
| **Complex I (2D – CI - ADP)** |  |  |  |  |  |  |  |
| Left ventricle | 47.206 | 20.532 | 52.548 | 7.805 | 30.776 | 14.156 | 0.099 |
| Diaphragm | 16.786 | 12.351 | 18.415 | 8.007 | 12.139 | 8.264 | 0.586 |
| *Soleus* | 13.861 | 2.944 | 11.446 | 5.528 | 9.889 | 3.289 | 0.335 |
| *Tibialis anterior* | 13.456 | 7.898 | 18.718 | 7.524 | 14.267 | 5.911 | 0.479 |
| **Complex I (RCR_ADP_ CI + ADP)** |  |  |  |  |  |  |  |
| Left ventricle | 3.973 | 2.071 | 4.167 | 0.951 | 3.555 | 1.254 | 0.817 |
| Diaphragm | 4.248 | 3.078 | 3.550 | 1.073 | 4.780 | 1.005 | 0.655 |
| *Soleus* | 5.061 | 2.645 | 4.426 | 1.356 | 5.005 | 1.115 | 0.852 |
| *Tibialis anterior* | 5.270 | 2.655 | 4.729 | 1.381 | 4.270 | 1.023 | 0.709 |
| **Complex I (2c - CI + ADP + Cyt c)** |  |  |  |  |  |  |  |
| Left ventricle | 75.140 | 29.068 | 83.444 | 33.742 | 38.256 | 31.195 | 0.092 |
| Diaphragm | 19.078 | 14.215 | 15.749 | 8.128 | 8.225 | 4.028 | 0.318 |
| *Soleus* | 18.096 | 4.828 | 17.796 | 13.394 | 11.528 | 6.936 | 0.463 |
| *Tibialis anterior* | 19.232 | 10.919 | 23.611 | 8.581 | 17.356 | 7.595 | 0.556 |
| **Complex I (3U - CI + ADP + Cyt c + CCCP)** |  |  |  |  |  |  |  |
| Left ventricle | 82.022 | 32.535 | 91.349 | 39.049 | 61.926 | 43.676 | 0.491 |
| Diaphragm | 24.562 | 17.682 | 22.850 | 11.629 | 17.201 | 13.435 | 0.709 |
| *Soleus* | 17.873 | 9.697 | 20.578 | 17.203 | 12.713 | 7.737 | 0.600 |
| *Tibialis anterior* | 20.219 | 12.338 | 21.366 | 5.576 | 17.339 | 6.542 | 0.757 |
| **Complex II (5S - CI and CII + ADP + Cyt c + CCCP)** |  |  |  |  |  |  |  |
| Left ventricle | 212.769 | 66.982 | 188.400 | 91.136 | 153.344 | 120.736 | 0.625 |
| Diaphragm | 54.841 | 38.879 | 35.482 | 27.538 | 34.338 | 34.844 | 0.579 |
| *Soleus* | 37.385 | 8.828 | 36.583 | 26.836 | 33.953 | 16.375 | 0.956 |
| *Tibialis anterior* | 58.369 | 33.202 | 33.541 | 12.000 | 28.182 | 16.028 | 0.116 |
| **Complex I + Complex II (Rot - CII + ADP + Cyt c + CCCP)** |  |  |  |  |  |  |  |
| Left ventricle | 139.571 | 42.322 | 103.538 | 61.258 | 33.230 ^b^ | 24.323 | **0.009** |
| Diaphragm | 30.870 | 18.623 | 16.474 | 12.308 | 9.655 | 20.421 | 0.072 |
| *Soleus* | 25.191 | 7.967 | 17.368 | 16.697 | 20.421 | 10.488 | 0.615 |
| *Tibialis anterior* | 18.998 | 13.599 | 19.195 | 6.980 | 10.849 | 6.163 | 0.321 |
| **Complex IV (AsTm) CIV + ADP + Cyt c + CCCP** |  |  |  |  |  |  |  |
| Left ventricle | 299.428 | 267.293 | 497.812 | 191.030 | 367.127 | 133.142 | 0.331 |
| Diaphragm | 70.254 | 62.706 | 162.731^a^ | 45.714 | 110.805 | 23.967 | **0.028** |
| *Soleus* | 43.895 | 36.225 | 125.916 | 20.278 | 156.857^b^ | 104.275 | **0.045** |
| *Tibialis anterior* | 80.309 | 83.474 | 233.655^a^ | 46.101 | 181.213 | 55.679 | **0.008** |

**Note** – a: p<0.05 vs CON; b: p<0.05 vs CON; c: p<0.05 vs POL; CON: control group; CS: citrate synthase; CSA: cross sectional area; DRP1: dynamin-related protein 1; g: grams; Km: kilometer; maximal phosphorylating respiration with electrons provided by complexes I and II [(5S) CI and CII + ADP + Cyt c + CCCP]; maximal phosphorylating respiration with electrons provided only by complex II [(7Rot) CII + ADP + Cyt c + CCCP]; maximal phosphorylating respiration with electrons provided only by complex IV [(AsTm) CIV + ADP + Cyt c + CCCP] maximal uncoupled mitochondrial respiration [(3U) CI + ADP + Cyt c + CCCP]; m: meters; MFN1: mitofusin 1; MFN2: mitofusin 2; NADH electron transfer-pathway state [(1PM) CI]; OD: optical density; OPA: optic atrophy protein 1; OXPHOS capacity [(2D) CI ADP]; PA: physical activity; PGC-1𝛼: peroxisome proliferator-activated receptor-gamma coactivator; POL: polarized training group; respiratory control ratio for ADP [(RCR_ADP_) CI + ADP]; respiratory flow after challenge with cytochrome c (2c) CI + ADP + Cyt c; TFAM: transcription factor A. mitochondrial; THR: threshold training group; TOM20: translocase of outer mitochondrial membrane 20; V̇O_2_max: maximal oxygen uptake; µm^2^ = square micrometer.
