# Supplementary material for: Polarized or threshold training: is there a superior training intensity distribution to improve V̇O2max, endurance capacity and mitochondrial function? A study in Wistar Rat models
Source: J Physiol Biochem. 2025 Apr 2;81(2):329–46. doi: 10.1007/s13105-025-01079-6 (PMC12279575; doi:10.1007/s13105-025-01079-6)
Supplement: Supplementary file 3 — Supplementary file3 (DOCX 15574 KB) [file 13105_2025_1079_MOESM3_ESM.docx]

**Online Resource S3**

**Title: Polarized or threshold training: is there a superior training intensity distribution to improve VO_2_max, endurance capacity and mitochondrial function? A study in Wistar Rat models**

**Journal:** Journal of Physiology and Biochemistry

**Authors:** *Oliveira, Pedro^1,2,3^; Anjos, Miguel^1, 2^; Flores, Ariane^1,2^;* Peixoto, Francisco^4^; Padrão, Ana Isabel^1,2^; *Fonseca, Hélder^1,2^*

*^1^Research Centre in Physical Activity, Health and Leisure (CIAFEL); Faculty of Sport of University of Porto (FADE-UP), Porto, Portugal*

*^2^Laboratory for Integrative and Translational Research in Population Health (ITR), Porto, Portugal*

^3^*Nucleus of Research in Human Motricity Sciences, Universidad Adventista de Chile, Chillán 3780000, Chile*

*^4^Vila Real Chemistry Center (CQVR), Biology and Environment Department, University of Trás-os-Montes and Alto Douro, 5000-801 Vila Real, Portugal*

**Corresponding author:** Pedro Oliveira ([up201807240@fade.up.pt](mailto:up201807240@fade.up.pt))

**Electronic Supplementary Material Appendix S3.** Loading control of Ponceau S staining of Mfn1, Mfn2, PGC-1𝛼, TFAM, DRP1, OPA1 and TOM20 for left myocardium ventricle, diaphragm, *soleus* and*, tibialis anterior.*


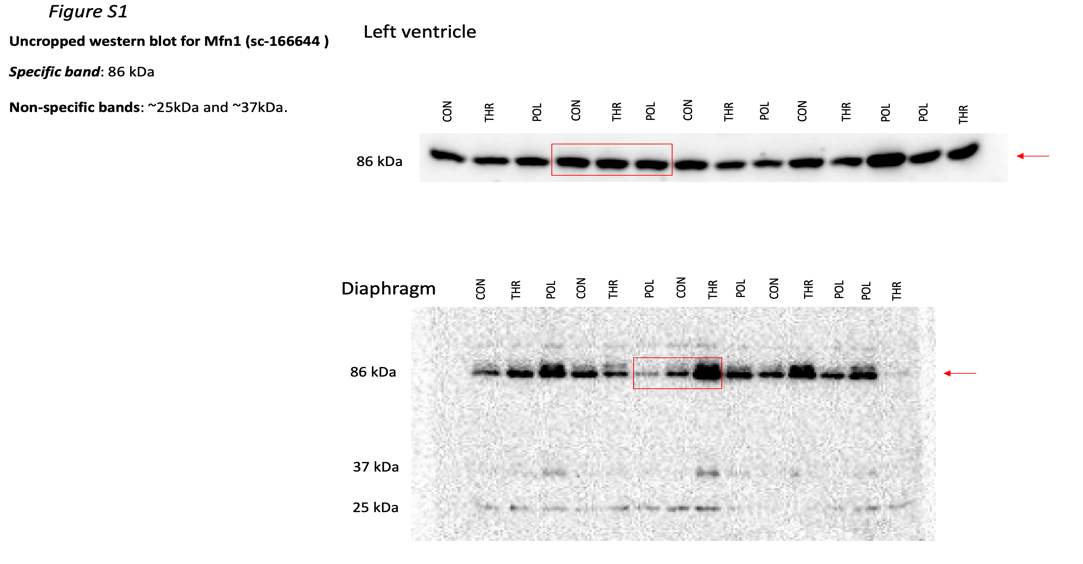

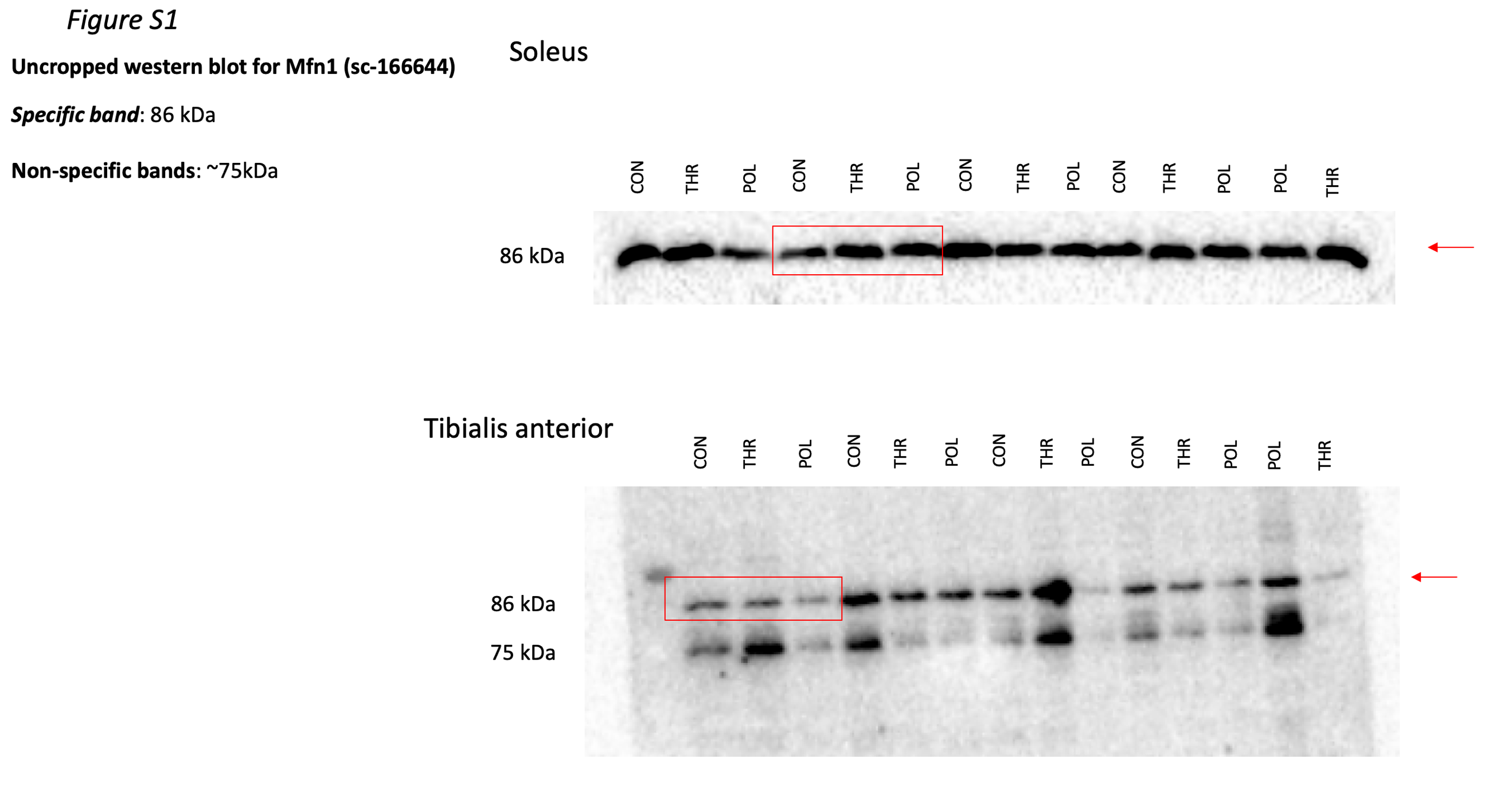


**
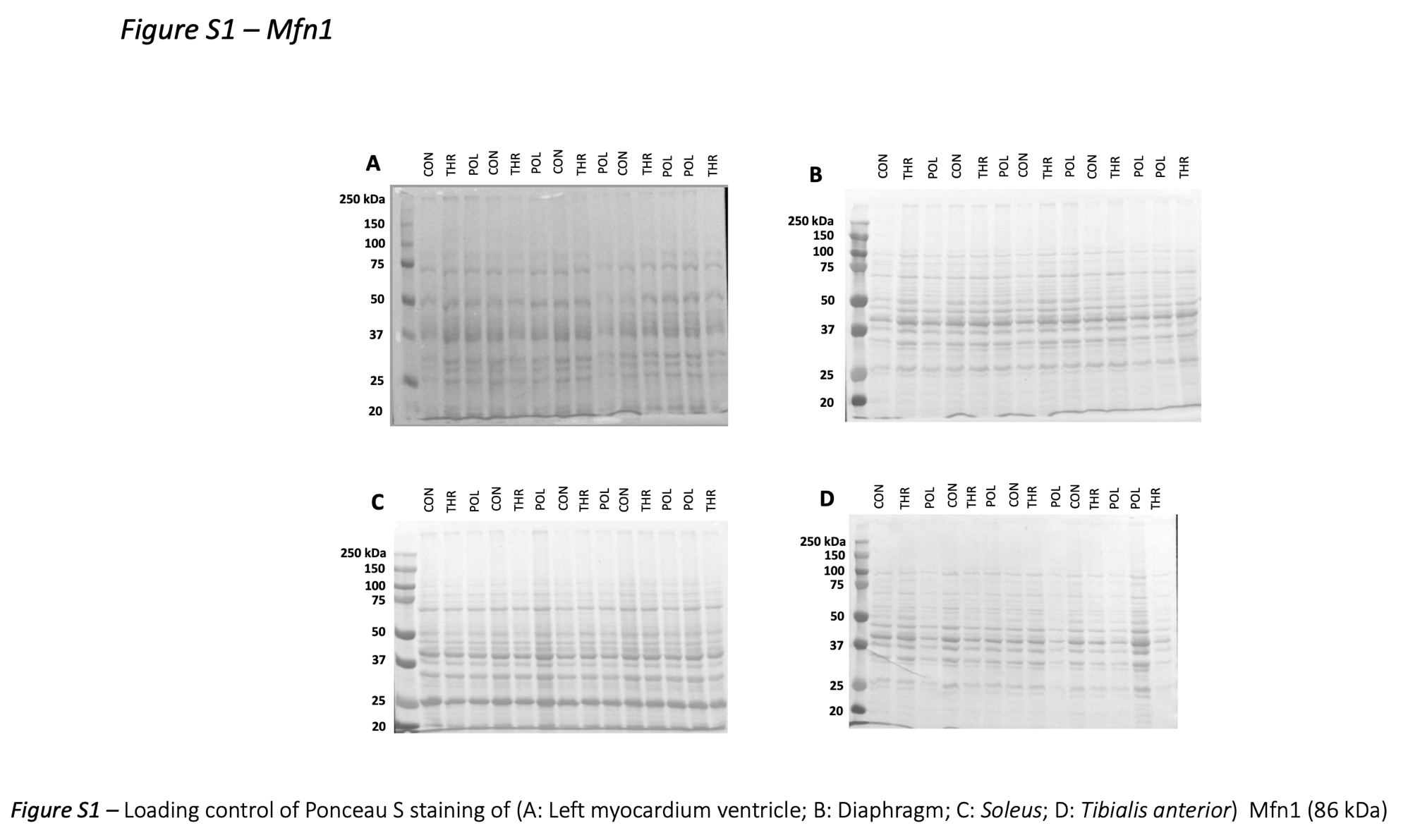
*Figure S1*** – Loading control of Ponceau S staining of (A: Left myocardium ventricle; B: Diaphragm; C: Soleus; D: Tibialis anterior) Mfn1 (86 kDa).


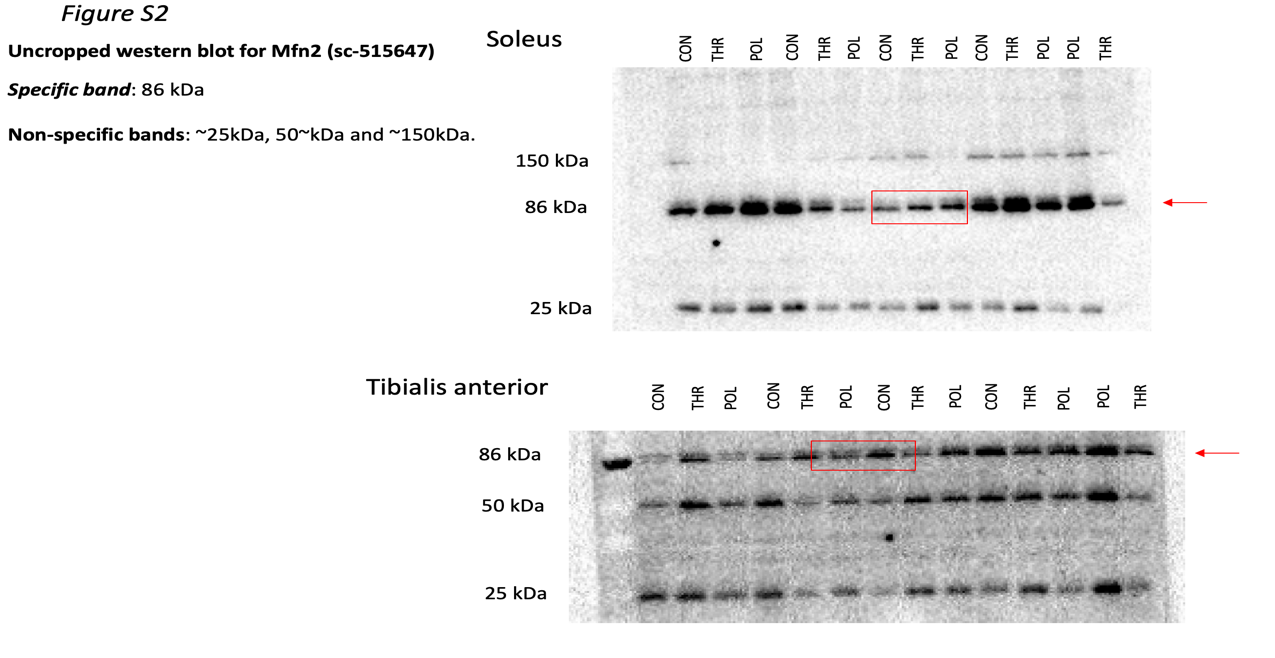

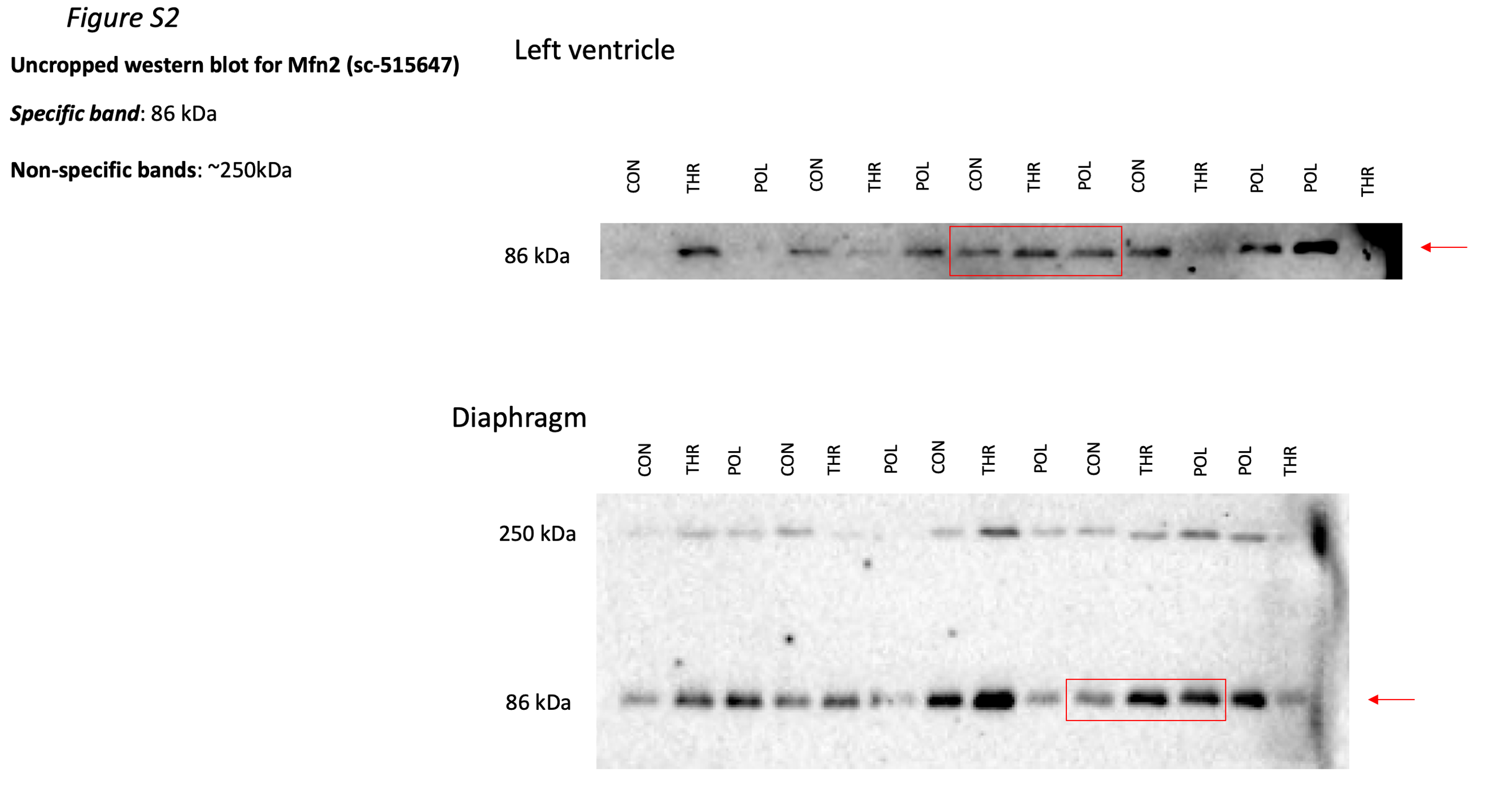


**
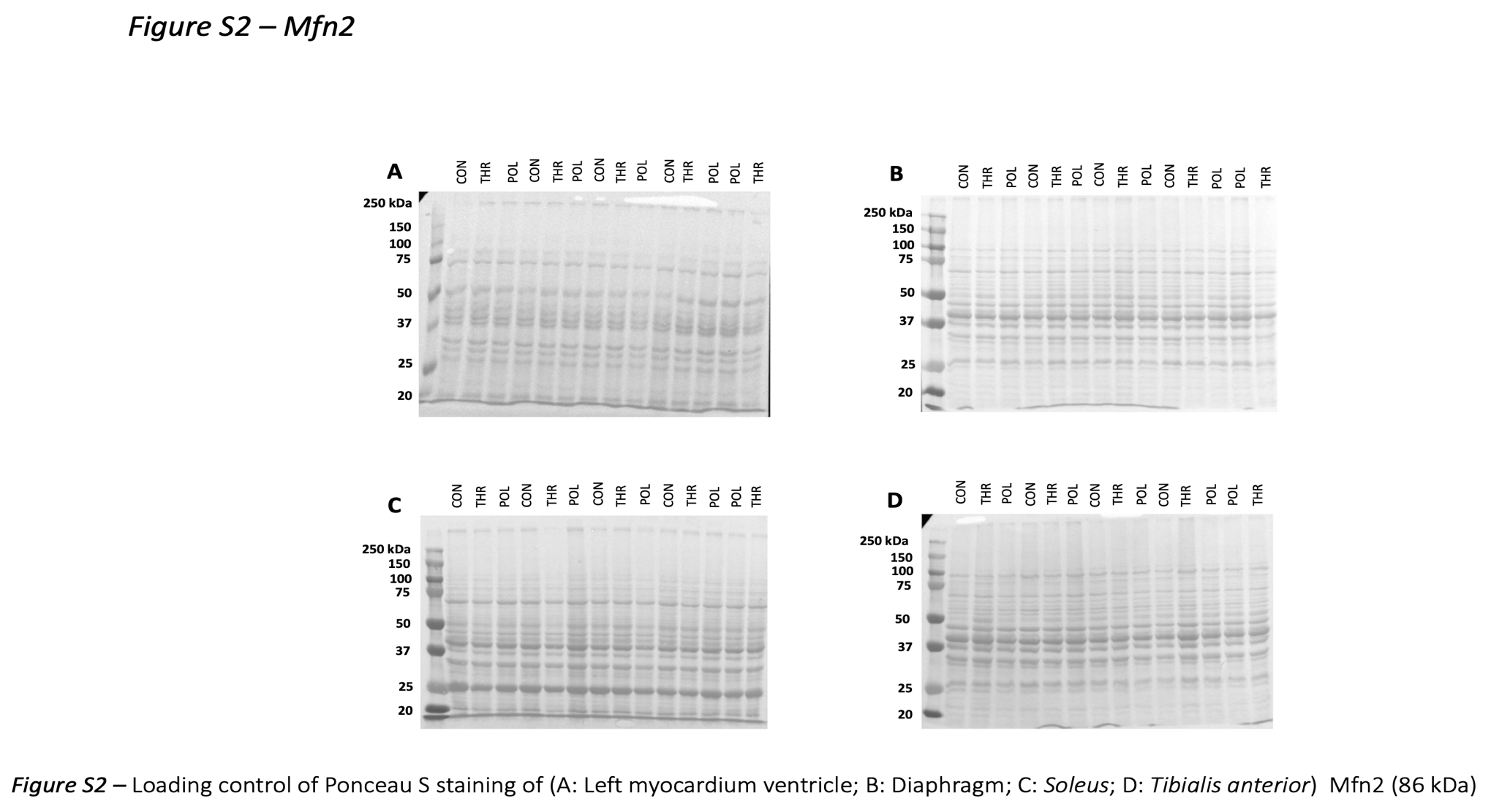
**

***Figure S2*** – Loading control of Ponceau S staining of (A: Left myocardium ventricle; B: Diaphragm; C: Soleus; D: Tibialis anterior) Mfn2 (86 kDa).


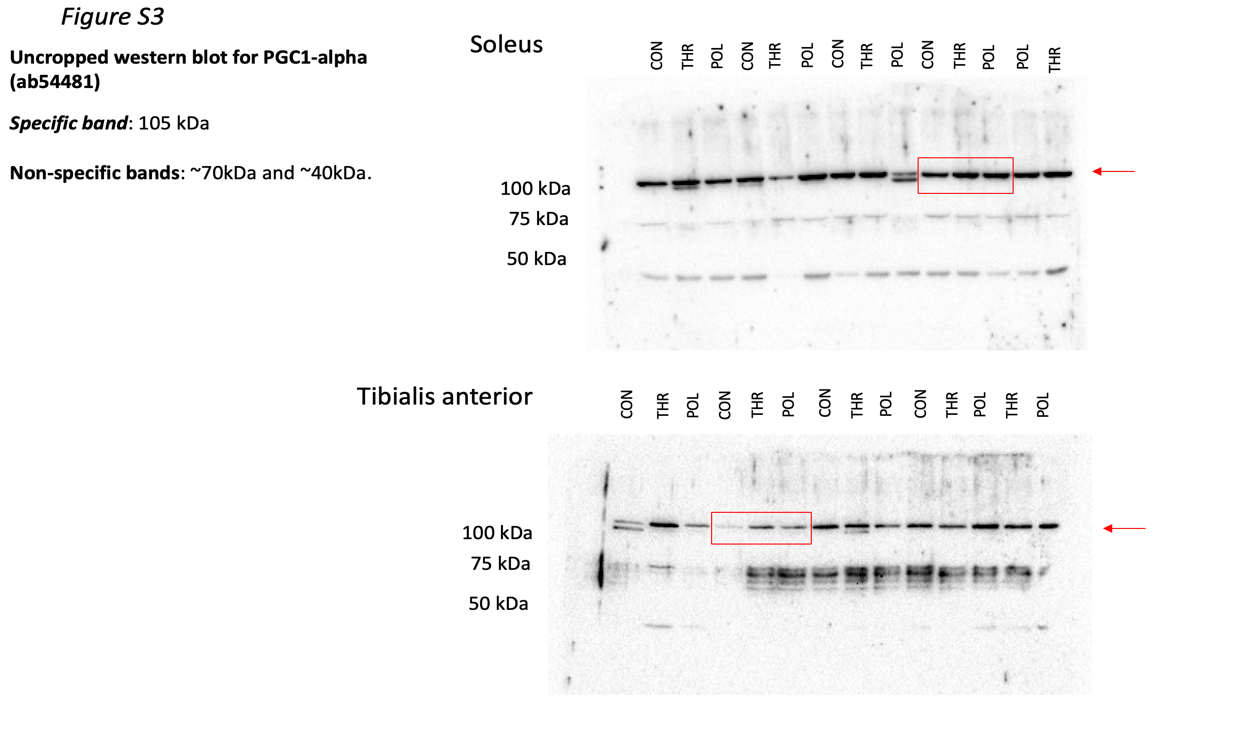

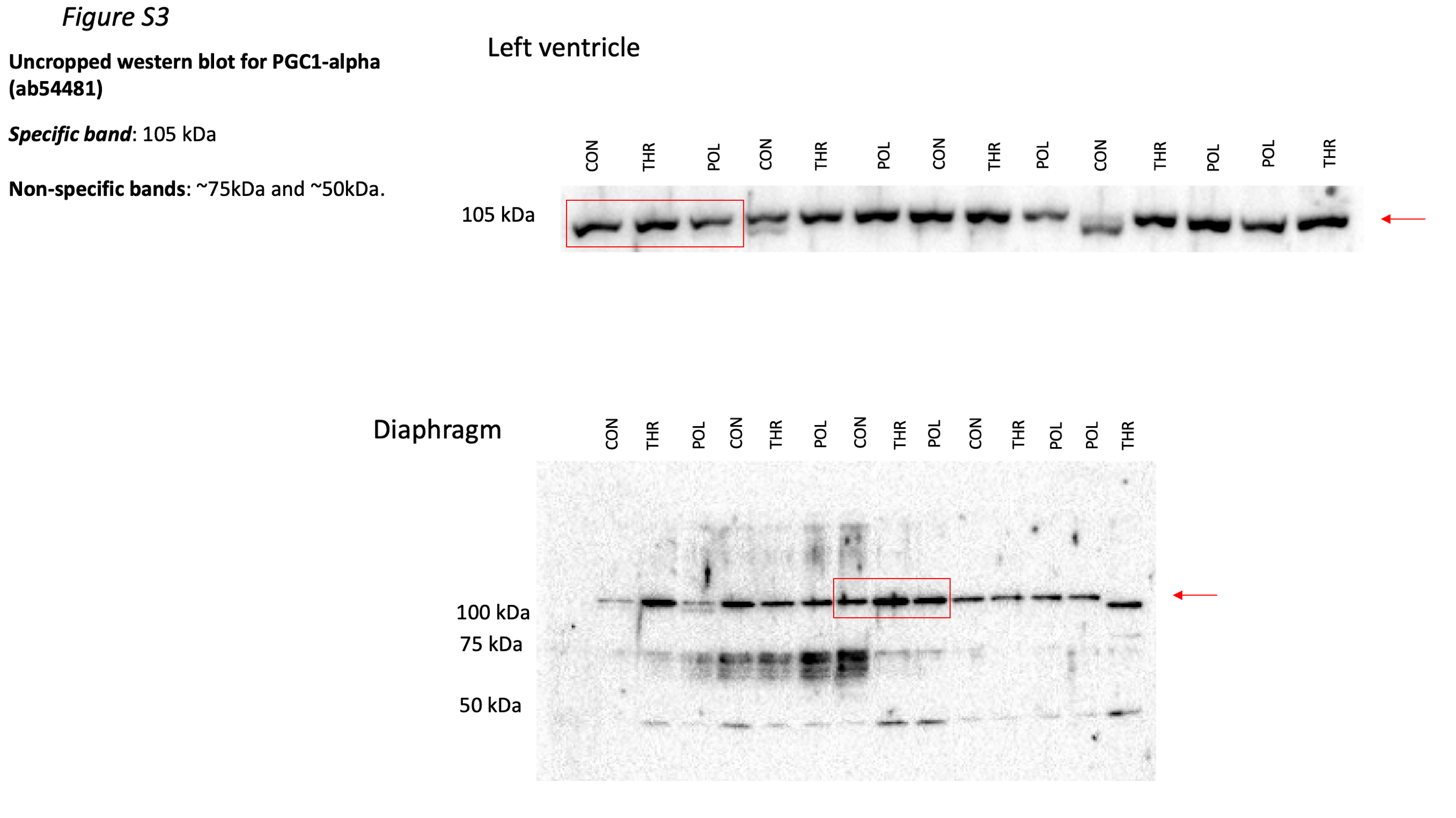


**
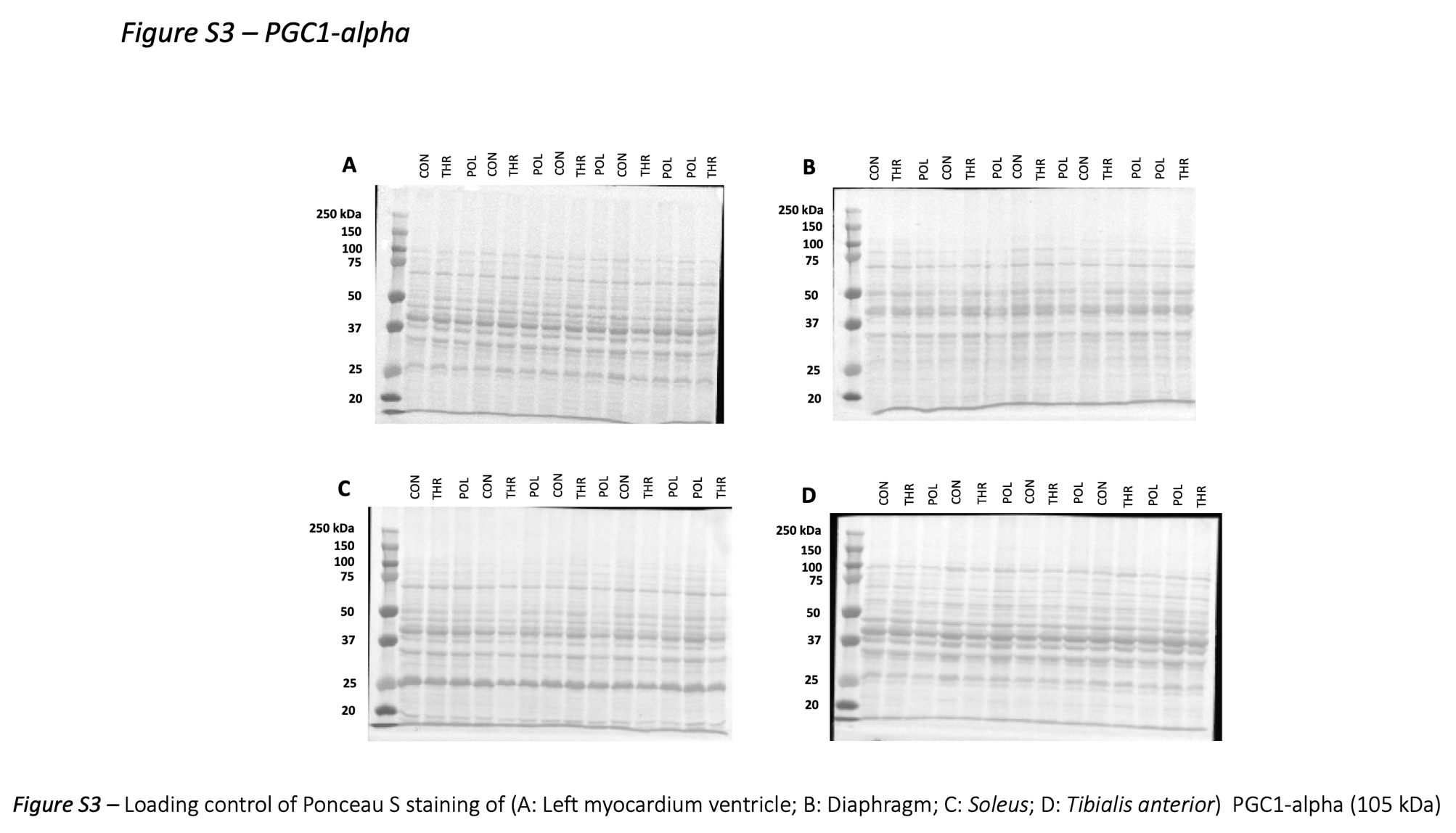
**

***Figure S3*** – Loading control of Ponceau S staining of (A: Left myocardium ventricle; B: Diaphragm; C: Soleus; D: Tibialis anterior) PGC-1𝛼 (105 kDa).

**
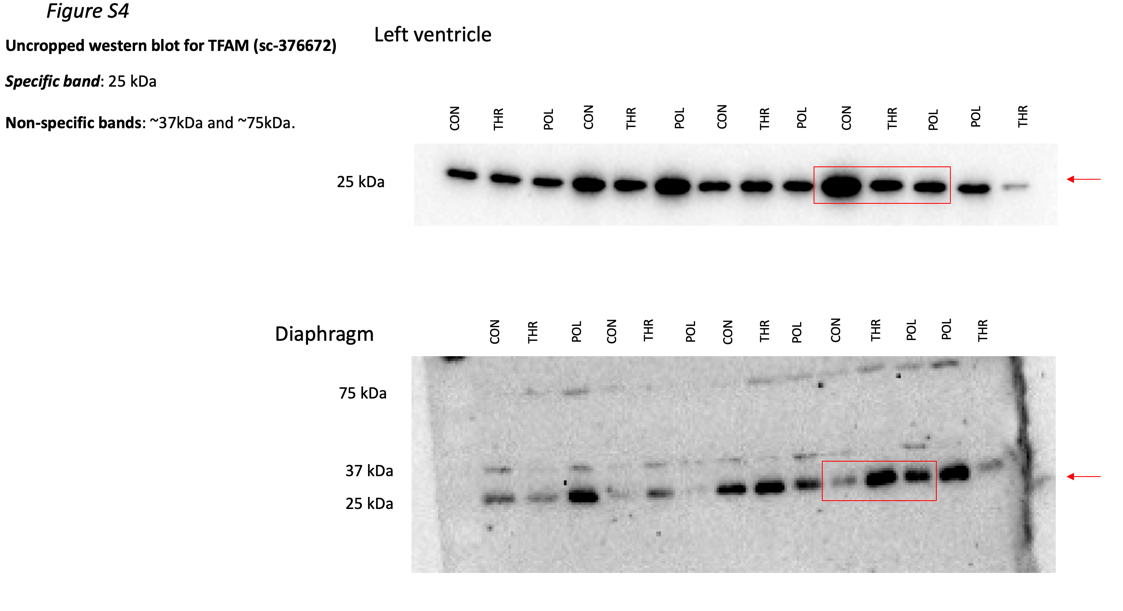
**


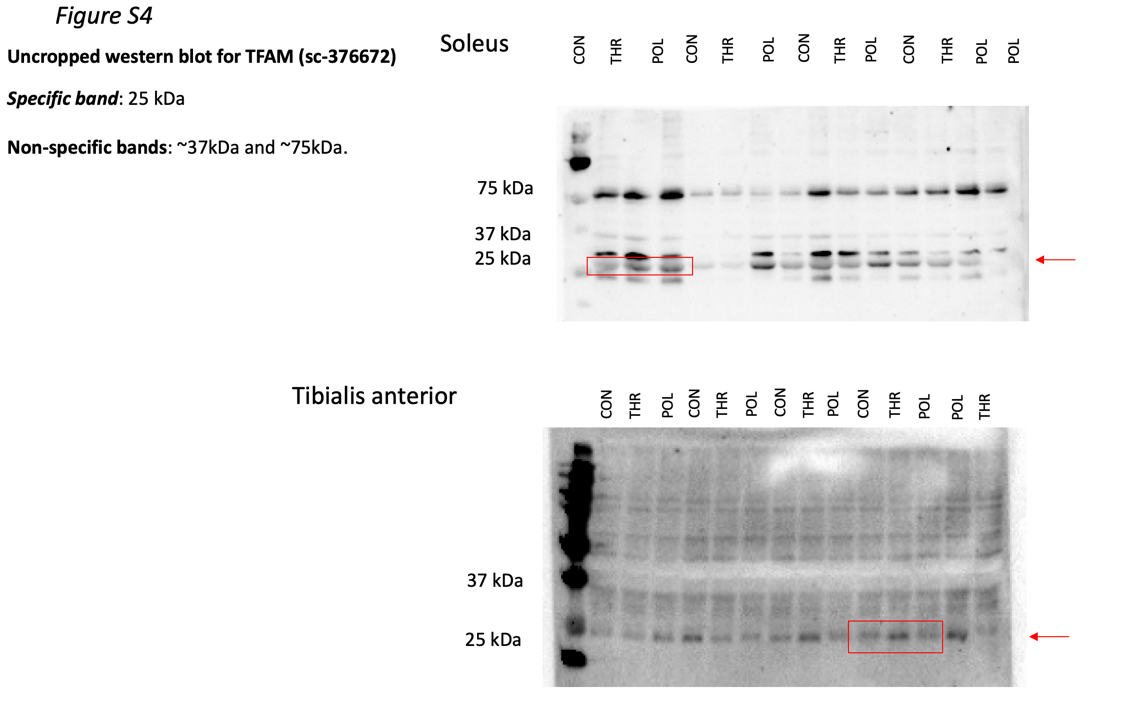


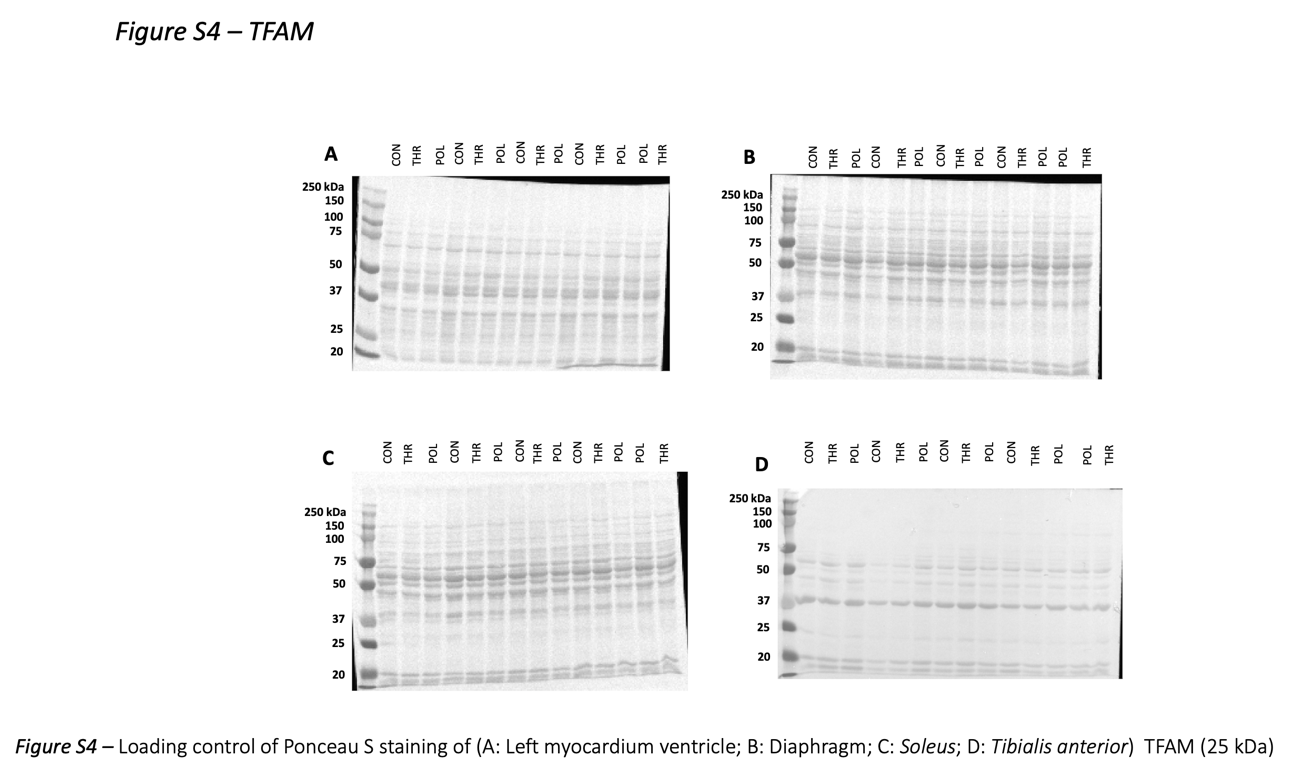


***Figure S4*** – Loading control of Ponceau S staining of (A: Left myocardium ventricle; B: Diaphragm; C: Soleus; D: Tibialis anterior) TFAM (25 kDa).

***Figure S5***

**Uncropped western blot for DRP1 (#8170)**

**Specific band:** 78-82 kDa

**Non-specific bands:** ~25kDa

Left Ventricle

Diaphragm


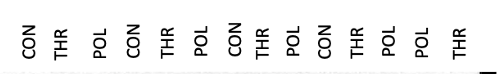

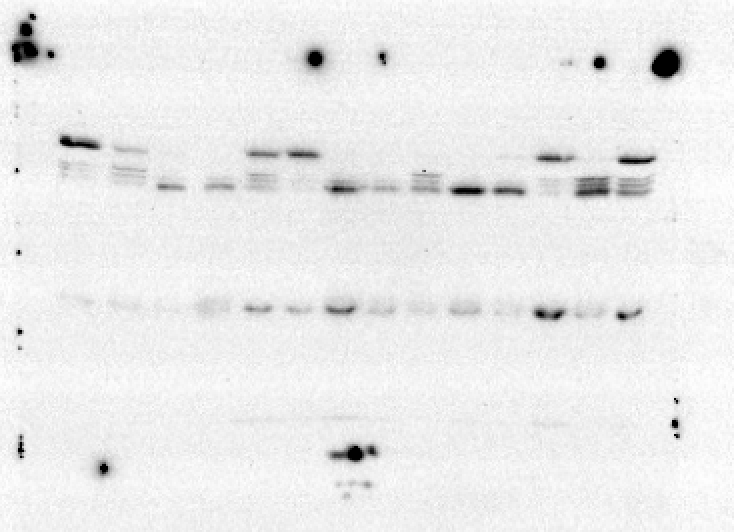

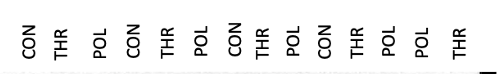

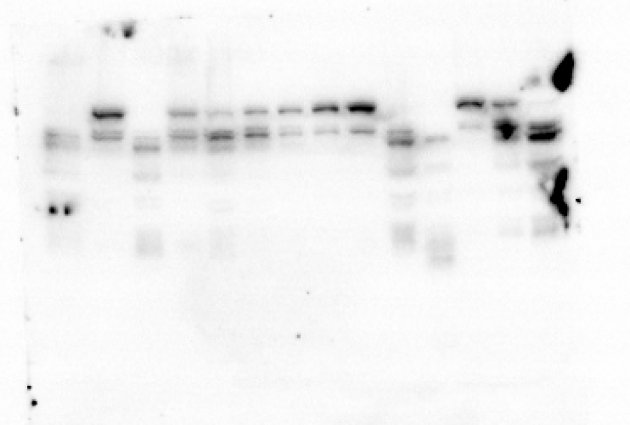


25kDa

78-82kDa

78-82kDa

Soleus

Tibialis anterior


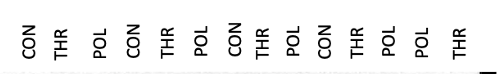

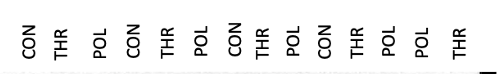


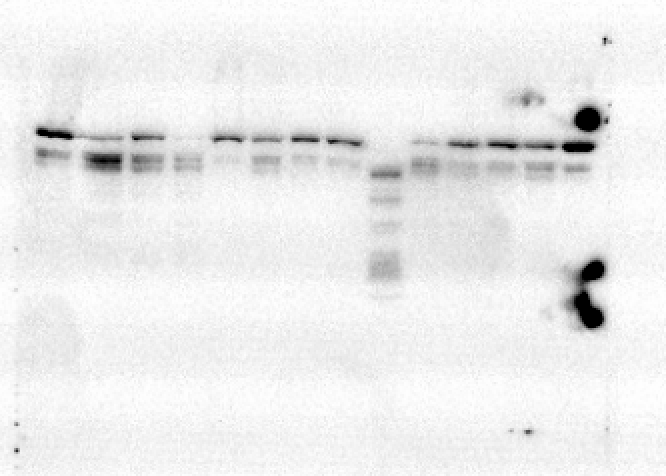

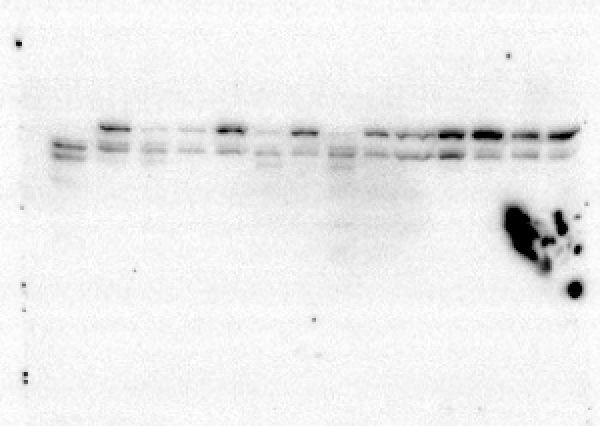


78-82kDa

78-82kDa


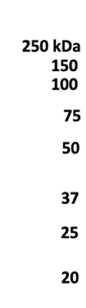

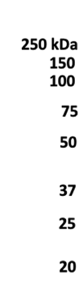

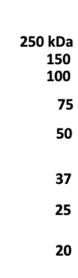

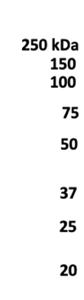

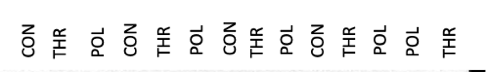

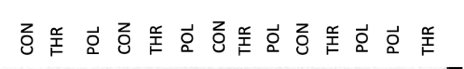

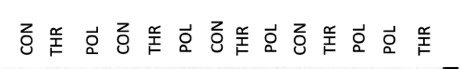

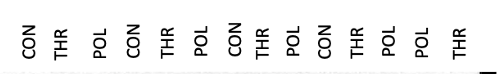

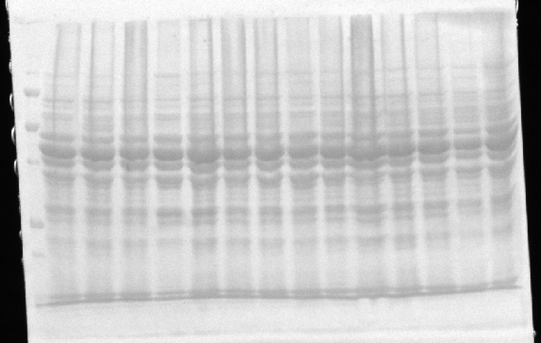

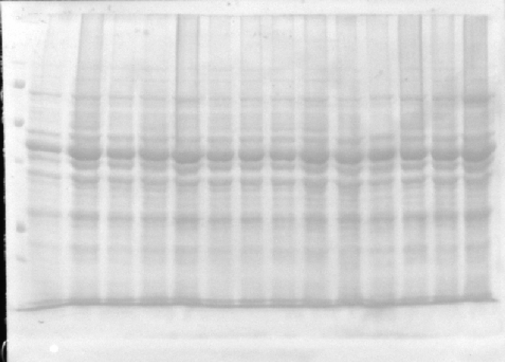

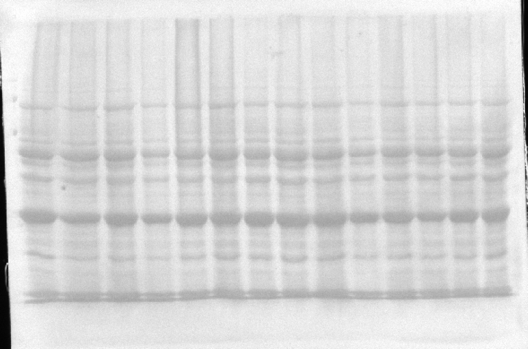

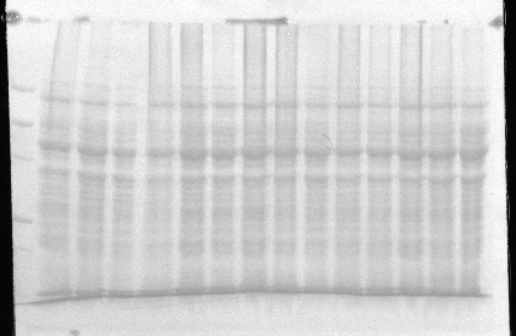


**D**

**B**

**A**

**C**

**D**

***Figure S5*** – Loading control of Ponceau S staining of (A: Left myocardium ventricle; B: Diaphragm; C: Soleus; D: Tibialis anterior) DRP1 (78-82 kDa).

***Figure S6***

**Uncropped western blot for OPA1 (ab119685)**

**Specific band:** 111 kDa

**Non-specific bands:** ~75kDa and ~37kDa

Diaphragm

Left Ventricle


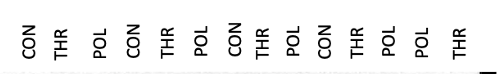

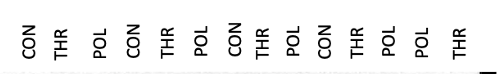

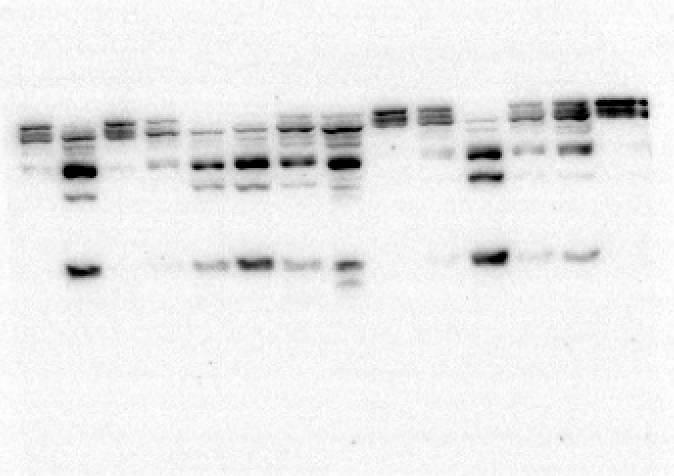


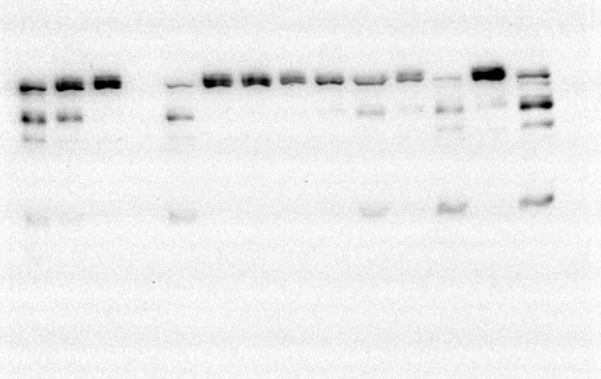


75kDa

111kDa

111kDa

37kDa

75kDa

37kDa

Soleus

Tibialis anterior


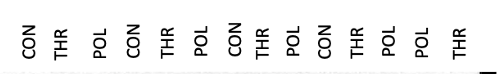

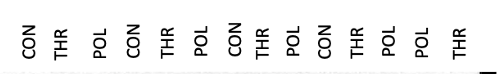


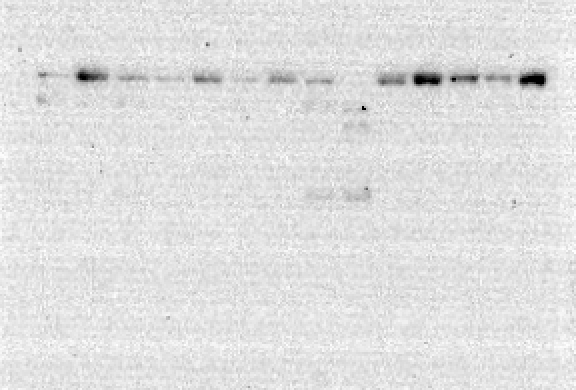

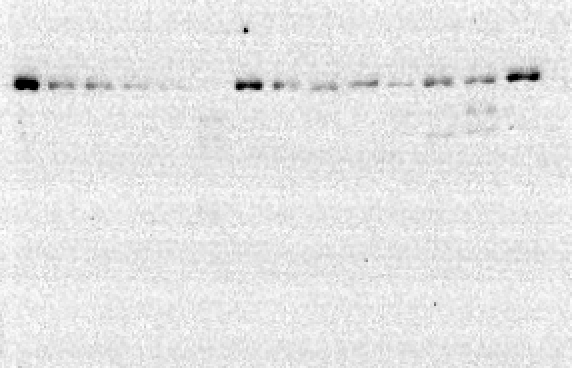


111kDa

111kDa


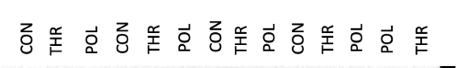

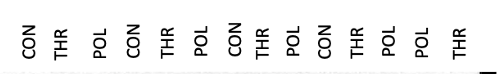


**A**

**B**


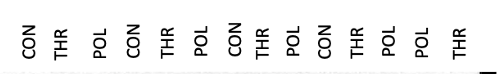

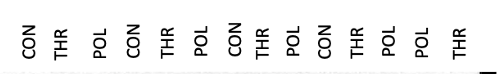

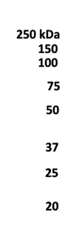

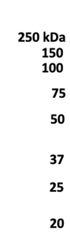

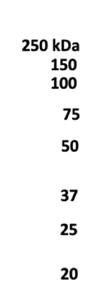

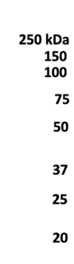

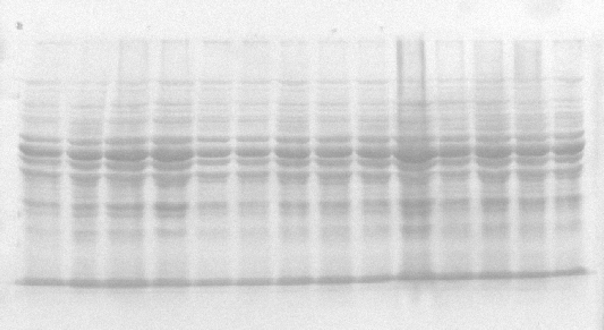

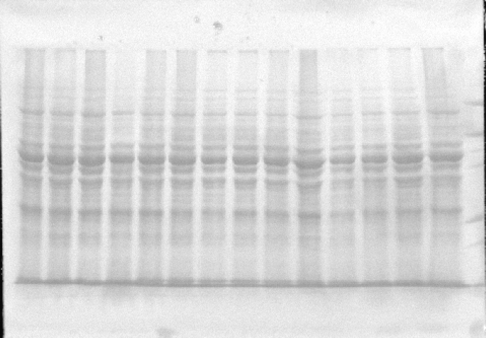

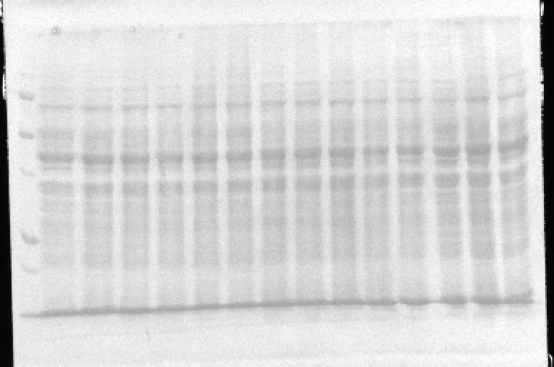

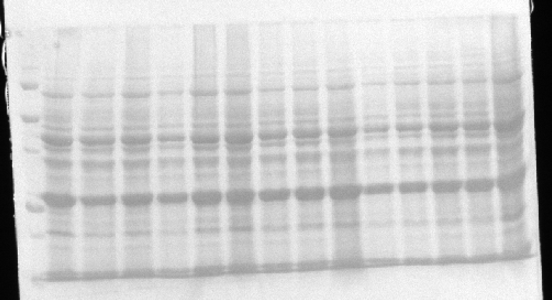


**D**

**C**

***Figure S6*** – Loading control of Ponceau S staining of (A: Left myocardium ventricle; B: Diaphragm; C: Soleus; D: Tibialis anterior) OPA1 (111kDa).

***Figure S7***

**Uncropped western blot for TOM20 (sc-11415)**

**Specific band:** 20 kDa

Left Ventricle

Diaphragm


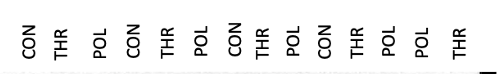

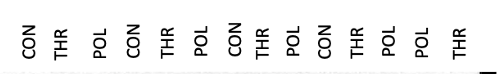


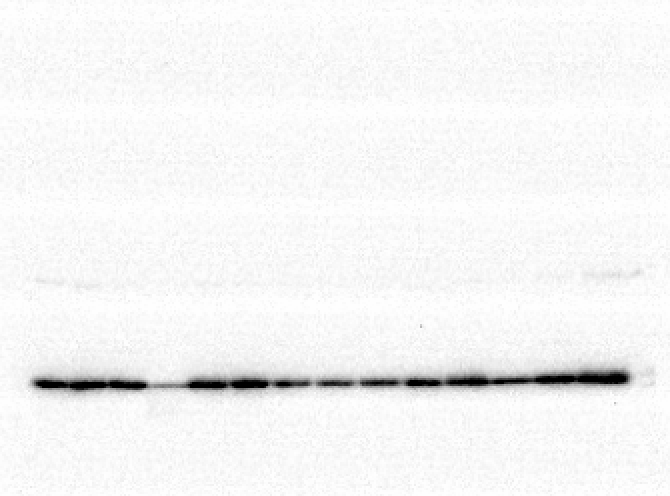

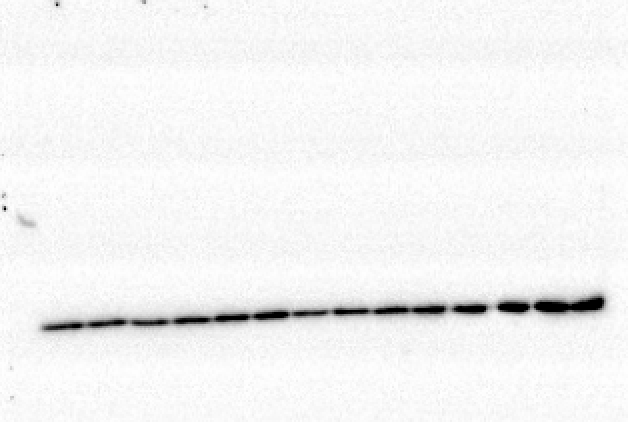


20kDa

20kDa

Tibialis anterior

Soleus


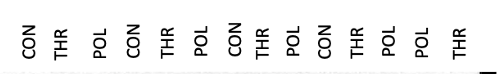


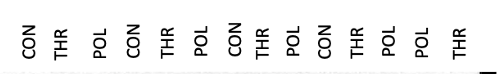


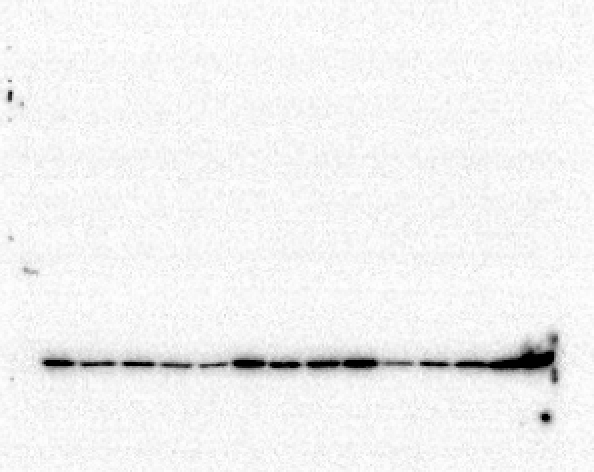


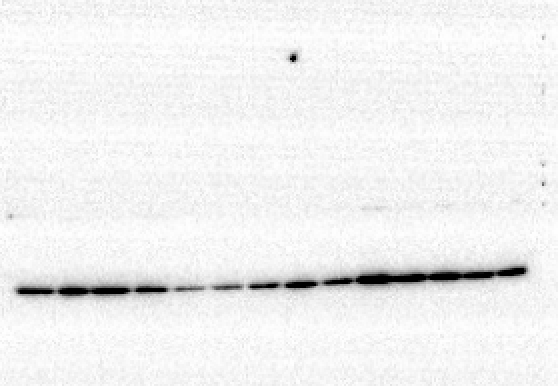


20kDa

20kDa


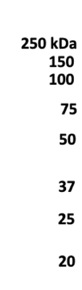

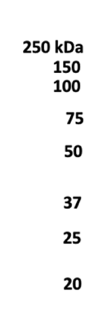

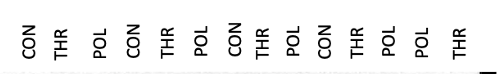

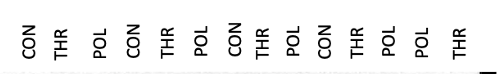


**B**

**A**


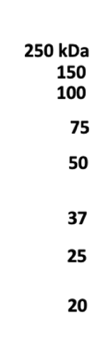

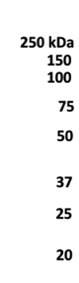

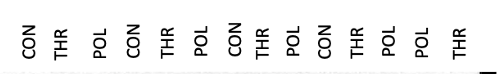

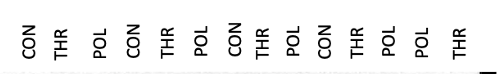

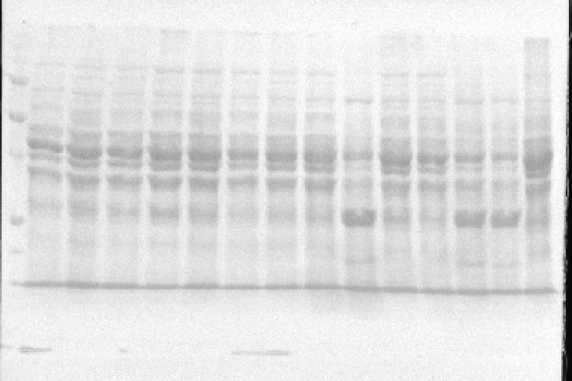

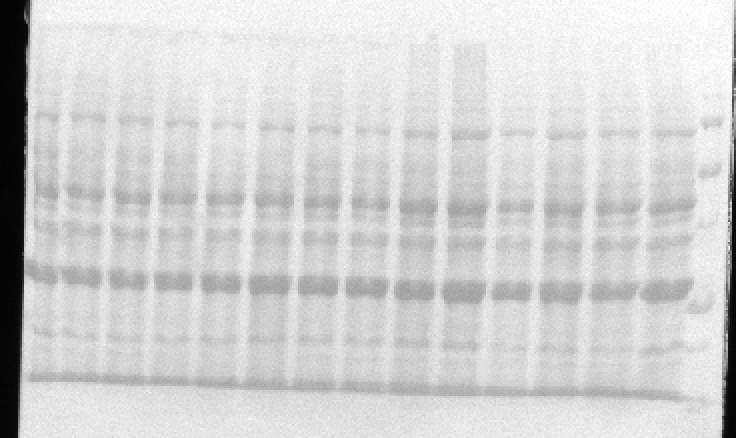

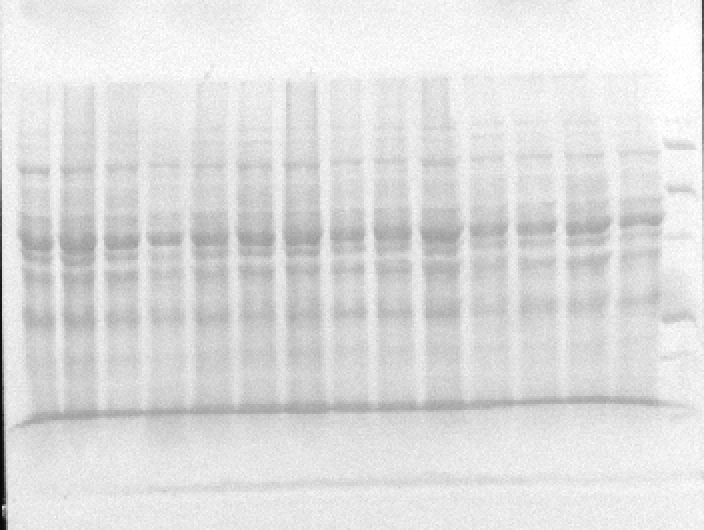

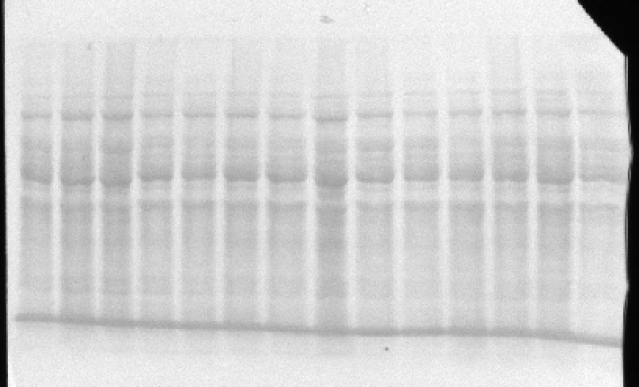


**C**

**D**

***Figure S7*** – Loading control of Ponceau S staining of (A: Left myocardium ventricle; B: Diaphragm; C: Soleus; D: Tibialis anterior) TOM20 (20 kDa).
